# Effects of spirulina supplementation alone or with exercise on cardiometabolic health in overweight and obese adults: a systematic review and meta-analysis

**DOI:** 10.3389/fnut.2025.1624982

**Published:** 2025-06-27

**Authors:** Zhenliang Fu, Shibiao Zhou, Xueyan Gu

**Affiliations:** School of Physical Education, Jiangxi Normal University, Nanchang, China

**Keywords:** spirulina, exercise, overweight, obesity, cardiometabolic health, meta-analysis

## Abstract

**Purpose:**

This systematic review and meta-analysis evaluates the effects of Spirulina supplementation, alone or combined with exercise, on body composition, lipid profiles, glycemic control, blood pressure, and cardiorespiratory health in overweight and obese adults. It also examines the moderating roles of participant characteristics and intervention protocols.

**Methods:**

We systematically searched PubMed, Embase, Cochrane Library, and Web of Science from their inception to September 2024. Results were pooled using random-effects models and reported as Hedge’s g (*g*) with 95% confidence intervals (95% CI). Heterogeneity was explored through subgroup and regression analyses. Bias risk and evidence quality were assessed using the Cochrane RoB 2 tool and the Grading of Recommendations Assessment, Development, and Evaluation (GRADE) approach.

**Results:**

Twenty-three studies (1,035 participants) were included. Spirulina supplementation alone significantly reduced body weight (*g* = −0.30, 95% CI: −0.53 to −0.08), total cholesterol (*g* = −0.79, 95% CI: −1.18 to −0.41), triglycerides (*g* = −0.64, 95% CI: −1.00 to −0.28), low-density lipoprotein cholesterol (LDL-C; *g* = −0.71, 95% CI: −1.13 to −0.29), and diastolic blood pressure (*g* = −0.73, 95% CI: −1.43 to −0.03), while increasing high-density lipoprotein cholesterol (HDL-C; *g* = 0.53, 95% CI: 0.04 to 1.02). When combined with exercise, Spirulina further improved HDL-C (*g* = 1.08, 95% CI: 0.37 to 1.79) and LDL-C (*g* = −0.81, 95% CI: −1.59 to −0.04). Subgroup and regression analyses revealed that participant age, body mass index, health status, Spirulina form, dosage, and intervention duration influenced outcomes.

**Conclusion:**

Spirulina may serve as a valuable adjunctive therapy for overweight and obese individuals with metabolic disorders, reducing cardiovascular risk by improving lipid profiles, blood pressure, and body weight. Combining Spirulina with exercise enhances certain lipid outcomes. However, its overall impact on body composition and glycemic control appears limited. Further research is needed to confirm its long-term efficacy.

**Systematic review registration:**

https://www.crd.york.ac.uk/PROSPERO/view/CRD42024573534, identifier CRD42024573534.

## 1 Introduction

Obesity poses a significant global public health challenge, markedly increasing the risk of metabolic diseases. In 2022, approximately 2.5 billion adults worldwide were overweight, with 890 million diagnosed as obese (1). Beyond weight gain and altered body composition, obesity substantially elevates the incidence and mortality of cardiometabolic diseases, including type 2 diabetes, hypertension, dyslipidemia, and atherosclerosis, placing a considerable burden on healthcare systems (2, 3). Although lifestyle interventions, such as dietary control and increased physical activity, remain central to obesity management, their long-term adherence is often challenging (4, 5). Consequently, research has increasingly focused on cost-effective and accessible dietary supplements or alternative therapies (6–8).

Spirulina, a commercial name for microalgae of the *Limnospira* genus (formerly *Arthrospira*), is widely recognized for its nutritional properties (9). Currently used as a dietary supplement or whole food, Spirulina primarily comprises *Arthrospira platensis* and *Arthrospira maxima*, the two main commercially produced species (9). Dried Spirulina typically contains 60–70% protein, 15–20% carbohydrates, 5–8% lipids, vitamins, minerals, essential fatty acids, β-carotene, and the rare essential γ-linolenic acid (10, 11). Due to its high protein and bioactive compound content, it is often referred to as a “food of the future” or “superfood.” Research suggests that Spirulina may regulate body weight and metabolic health by inhibiting macrophage migration to visceral fat, reducing hepatic fat accumulation, lowering oxidative stress, enhancing insulin sensitivity, and increasing satiety (10, 12).Both animal studies (13–15) and human clinical trials (12, 16, 17) report benefits for weight management, lipid profiles, and blood glucose regulation. Previous systematic reviews and meta-analyses (18–22) further indicate that Spirulina supplementation may reduce body weight and improve lipid profiles, blood pressure, and glucose levels, although findings remain inconsistent.

For instance, Bohórquez-Medina et al. (23) found no significant improvements in blood glucose or certain lipid markers in patients with obesity-related metabolic disorders following Spirulina supplementation, while Hamedifard et al. (24) reported no notable changes in triglyceride levels among individuals with metabolic syndrome. These findings suggest variability in Spirulina’s efficacy across populations. Thus, further research is needed to clarify its effects in overweight and obese adults. Moreover, prior studies have not thoroughly explored how participant characteristics (e.g., age, BMI, health status) or intervention parameters (e.g., dose, form, duration) modulate outcomes. Notably, optimal dosing and intervention duration remain unclear, hindering the development of standardized Spirulina protocols.

Additionally, exercise, a cornerstone of obesity management, has been increasingly studied in combination with Spirulina. Some evidence suggests that their combined use may synergistically improve body composition and metabolic health in overweight and obese individuals (25, 26). However, Golestani et al. (27) found that combining high-intensity interval training with Spirulina did not improve lipid profiles in overweight and obese women. This underscores the need for a systematic meta-analysis to evaluate the combined effects of Spirulina supplementation and exercise.

This systematic review and meta-analysis aims to comprehensively evaluate the effects of Spirulina supplementation, alone or combined with exercise, on cardiometabolic health in overweight and obese adults. By analyzing moderating factors—such as participant age, BMI, health status, and Spirulina dose, form, and intervention duration—this study seeks to establish optimal Spirulina protocols and explore potential synergistic effects with exercise. Ultimately, it aims to provide precise, evidence-based guidance for managing metabolic health in overweight and obese populations.

## 2 Methods

### 2.1 Registration

This systematic review and meta-analysis adhered to the Preferred Reporting Items for Systematic Reviews and Meta-Analyses (PRISMA) guidelines (28) and was pre-registered in PROSPERO (ID: CRD42024573534).

### 2.2 Search strategy

We systematically searched PubMed, Embase, Cochrane Library, and Web of Science from their inception to September 7, 2024. The PubMed search used the terms: (“Spirulina” OR “Arthrospira” OR “Spirulina platensis” OR “Spirulina maxima” OR “Arthrospira platensis” OR “Arthrospira maxima”) AND (“overweight” OR “obese” OR “obesity” OR “hypertension” OR “diabetes” OR “metabolic syndrome” OR “body mass index” OR “BMI” OR “body weight” OR “body composition” OR “anthropometric indices” OR “blood pressure” OR “lipids” OR “glucose” OR “fasting blood glucose” OR “FBG” OR “insulin” OR “triglyceride” OR “TG” OR “low density lipoprotein cholesterol” OR “LDL-C” OR “high density lipoprotein cholesterol” OR “HDL-C” OR “total cholesterol” OR “TC”). Search strategies were tailored for other databases by adjusting syntax accordingly. Reference lists of relevant meta-analyses and articles were manually screened to identify additional studies. Two authors (Fu and Zhou) independently reviewed titles and abstracts, with disagreements resolved by a third author (Gu).

### 2.3 Eligibility criteria

Inclusion criteria followed the PICOS framework (Population, Intervention, Comparison, Outcome, Study design):

Population: Adults aged > 18 years with a BMI > 25 kg/m^2^, regardless of gender or comorbidities (e.g., type 2 diabetes, hypertension).

Intervention: Spirulina supplementation (alone or with exercise) for ≥ 2 weeks, delivered as tablets, capsules, or powder.

Comparison: Placebo, control group, exercise alone, or exercise + placebo.

Outcome: At least one measure of metabolic health or body composition, including body weight (BW), body mass index (BMI), body fat percentage (BFP), waist circumference (WC), waist-to-hip ratio (WHR), systolic blood pressure (SBP), diastolic blood pressure (DBP), maximal oxygen uptake (VO2max), total cholesterol (TC), triglycerides (TG), high-density lipoprotein cholesterol (HDL-C), low-density lipoprotein cholesterol (LDL-C), fasting blood glucose (FBG), or insulin (INS).

Study Design: Randomized or non-randomized controlled trials with parallel or crossover designs.

Exclusion criteria encompassed non-human studies, non-English publications, interventions < 2 weeks, those involving other supplements or medications, and studies without metabolic or body composition outcomes. Two authors (Fu and Zhou) independently evaluated full-text eligibility, with disagreements resolved by a third author (Gu).

### 2.4 Data extraction and conversion

Two authors (Fu and Zhou) independently extracted data, with accuracy verified by a third author (Gu). Extracted information included: first author, publication year, region, study design, participant characteristics (age, BMI, health status), supplementation protocol (dose, form, duration), exercise protocol (if applicable), and outcome measures (e.g., BW, BMI, TC) with means (M) and standard deviations (SD). All outcomes were standardized to consistent units: BW (kg), BMI (kg/m^2^), BFP (%), WC (cm), TC (mg/dL), TG (mg/dL), HDL-C (mg/dL), LDL-C (mg/dL), FBG (mg/dL), INS (μIU/mL), SBP (mmHg), DBP (mmHg), and VO2max (mL/kg/min). For missing data, study authors were contacted; studies were excluded if no response was received.

For each group’s pre- and post-intervention data(M, SD, and sample size), the mean difference (MD) was calculated as follows (29):


$$M\,D_{d\,i\,f\,f}=M_{p\,o\,s\,t}-M_{p\,r\,e}$$

where *M*_*post*_ is the post-intervention mean and *M*_*pre*_ is the pre-intervention mean. If standard error (SE) was reported instead of SD, it was converted to SD using the formula:


$$S\,D=S\,E\times \sqrt{N}$$

where N is the group sample size. The SD of the mean difference (*SD*_*diff*_) was then calculated as (29):


$$S\,D_{d\,i\,f\,f}=\sqrt{S\,D_{p\,r\,e}^{2}+S\,D_{p\,o\,s\,t}^{2}-2\,r\times S\,D_{p\,r\,e}\times S\,D_{p\,o\,s\,t}}$$

where *SD*_*pre*_ is the pre-intervention SD, *SD*_*post*_ is the post-intervention SD, and *r* is the correlation coefficient between pre- and post-intervention measurements. As the original studies did not report the correlation coefficient (r), we assumed *r* = 0.8 based on similar studies (30–34).

### 2.5 Risk of bias and evidence quality

Risk of bias was evaluated using the Cochrane Risk of Bias 2.0 tool (August 2019) (35), assessing five domains: randomization process, deviations from intended interventions, missing outcome data, outcome measurement, and reported result selection. Two authors (Fu and Zhou) independently assessed each study, with discrepancies resolved by a third author (Gu). Studies were rated as having “low,” “some concerns,” or “high” overall bias.

Evidence quality was assessed using the Grading of Recommendations Assessment, Development, and Evaluation (GRADE) framework, categorized as “high,” “moderate,” “low,” or “very low.” Downgrading occurred for risk of bias (“some concerns” or “high” prompted a one-level downgrade), inconsistency (I^2^ > 50% prompted a one-level downgrade), imprecision (statistical power < 80% with unclear effect direction prompted a one-level downgrade), and publication bias [Egger’s test (36) *p* < 0.05 prompted a one-level downgrade]. GRADE assessments were performed by Fu and verified by Zhou.

### 2.6 Statistical analysis

Analyses were conducted using R (version 4.2.0) with the meta and metafor packages. Pooled effect sizes were estimated using a random-effects model [DerSimonian-Laird method (37)] and reported as Hedges’ g (*g*), classified as trivial (< 0.2), small (0.2–0.5), moderate (0.5–0.8), or large (> 0.8) (38). Results included 95% confidence intervals (CI) and prediction intervals (PI, t-distribution-based) to account for heterogeneity (39). Heterogeneity was evaluated with the I^2^ statistic: 0–25% (low), 25–75% (moderate), > 75% (high). Studies with CI not overlapping the pooled effect CI were flagged as outliers. Sensitivity analyses used the leave-one-out method, with statistical power assessed via the metameta package.

Heterogeneity sources were explored through subgroup and regression analyses, requiring ≥ 10 studies for regression and ≥ 5 studies per subgroup (40). Subgroup variables included age (18–44, 45–59, > 60 years), baseline BMI (25–30, > 30 kg/m^2^), health condition (e.g., type 2 diabetes, hypertension), Spirulina form (tablet, capsule, powder), dose (< 2, 2, 4–10 g/day), and duration (≤ 8, ≥ 12 weeks). Regression analyses employed linear (age, BMI, dose, duration) and cubic polynomial (dose, duration) models, visualized with ggplot2. Publication bias was examined using funnel plots (41) and Egger’s test (36), with *p* < 0.05 indicating significance. Statistical significance was set at *p* < 0.05, with 0.05 < *p* < 0.10 considered a trend.

## 3 Results

### 3.1 Search results

**FIGURE 1:**
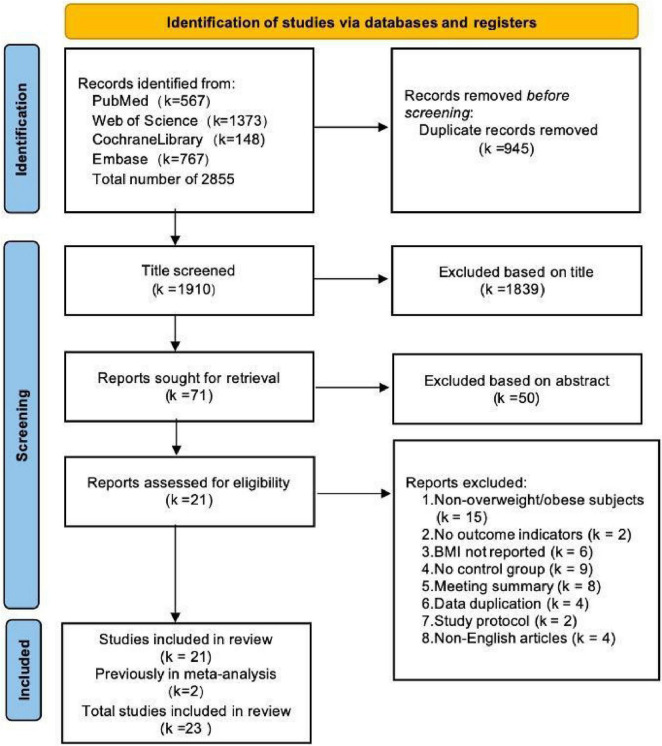
PRISMA flow diagram of study selection.

The database search across PubMed, Embase, Cochrane Library, and Web of Science retrieved 2,855 records. After removing 945 duplicates and excluding 1,889 records that failed to meet inclusion criteria during title and abstract screening, 23 studies (25–27, 42–61) (comprising 28 trials) were included. Of these, 21 trials (25, 26, 42–55, 57–61) examined Spirulina supplementation alone (Spirulina vs. control), while 7 trials (25–27, 42, 56, 59, 60) assessed Spirulina combined with exercise (Spirulina + exercise vs. exercise alone). The selection process is depicted in the PRISMA flow diagram (Figure 1).

### 3.2 Study characteristics

A total of 23 studies (28 trials) included 1,035 overweight or obese adults (BMI 25–40 kg/m^2^, aged 21–65 years, 430 males, 270 females, 335 with unreported gender). Of these, 21 trials (867 participants, BMI 25–40 kg/m^2^, aged 26–65 years) evaluated Spirulina supplementation alone, with intervention durations of 2–24 weeks and Spirulina doses of 0.02–10 g/day, administered as tablets, capsules, powders, pills, or extracts. Participants had conditions such as type 2 diabetes, hypertension, metabolic syndrome, treatment-naïve HIV on antiretroviral therapy, or ulcerative colitis. 7 trials (168 participants, BMI 28–33 kg/m^2^, aged 21–62 years) assessed Spirulina combined with exercise, with intervention durations of 4–12 weeks and Spirulina doses of 1–6 g/day, delivered as capsules, tablets, or powders. Exercise regimens included aerobic + resistance training (25, 26), high-intensity interval training (27, 56, 59), weighted yoga (60), or circuit resistance training (42), with participants being overweight or obese without other comorbidities. Control groups received placebo, no intervention, or placebo + exercise. Study details are presented in Tables 1, 2.

**TABLE 1:** Characteristics of included studies on the effects of Spirulina supplementation alone.

Study	Country	Design	Sample size	Participant details	Duration (weeks)	Supplementation protocol	Outcomes
Mani et al. (43)	India	CT	22 (50% male; SP: 15, CON: 7)	T2DM; Age: 49.6 ± 8.6; BMI: 28.1 ± 3.6	8	Spirulina tablet (2 g/day)	TC ↓; TG ↓; HDL-C↔; LDL-C ↓; FBG ↓
Parikh et al. (44)	India	RCT	25 (60% male; SP: 15, CON: 10)	T2DM; Age: 54.1 ± 6.4; BMI: 25.2 ± 4.4	8	Spirulina tablet (2 g/day)	TC↓; TG↓; HDL-C↑; LDL-C↓; FBG↓
Ngo-Matip et al. (45)	Cameroon	RCT/SB	159 (SP: 80, CON: 79)	HIV-ART-naïve; Age: 35.7 ± 9.7; BMI: 25.7 ± 4.8	24	Spirulina powder (10 g/day)	BMI↔; TC↓; TG↓; HDL-C↑; LDL-C↓; FBG↔
Jensen et al. (46)	USA	RCT/DB/PC	24 (21% male; SP: 12, PLA: 12)	Overweight/obese; Age: 46.2 ± 11.5; BMI: 28.8 ± 2.9	2	Spirulina capsule (2.3 g/day); Placebo: rice flour	SBP↔; DBP↔
Miczke et al. (47)	Poland	RCT/DB/PC	80 (51% male; SP: 40, PLA: 40)	Overweight/HTN; Age: 53.3 ± 5.6; BMI: 26.3 ± 3.2	12	Spirulina capsule (2 g/day); Placebo: microcrystalline cellulose	BW↓; BMI↓; SBP↓; DBP↔
Park and Lee, (48)	South Korea	RCT/DB/PC	33 (SP: 16, PLA: 17)	Overweight/obese; Age: 65.3 ± 4.0; BMI: 27.0 ± 2.1	16	Spirulina (8 g/day); Placebo: starch	TC↔; TG↔; HDL-C↔; LDL-C↔
Szulinska et al. (49)	Poland	RCT/DB/PC	50 (50% male; SP: 25, PLA: 25)	Obese/HTN; Age: 49.8 ± 7.9; BMI: 33.4 ± 6.4	12	Spirulina capsule (2 g/day); Placebo: microcrystalline cellulose	BW↓; BMI↓; WC↓; TC↓; TG↔; HDL-C↔; LDL-C↓; FBG↔; INS↔
Zeinalian et al. (50)	Iran	RCT/DB/PC	56 (16% male; SP: 29, PLA: 27)	Obese; Age: 34.4 ± 8.2; BMI: 33.0 ± 3.0	12	Spirulina tablet (1 g/day); Placebo: starch without chlorophyll	BW↓; BMI↓; WC↔; TC↓; TG↔; HDL-C↔; LDL-C↔
Hernández-Lepe et al. (26)	Mexico	RCT/DB/PC Crossover	24 (100% male; SP: 12, PLA: 12)	Overweight/obese; Age: 26 ± 5; BMI: 30.9 ± 4.9	12 (6 × 6)	Spirulina capsule (4.5 g/day); Placebo: low-calorie saccharin powder	BMI↔; BFP↓
Yousefi et al. (51)	Iran	RCT/DB/PC	38 (18% male; SP: 19, PLA: 19)	Overweight/obese; Age: 40.0 ± 9.5; BMI: 32.8 ± 4.3	12	Spirulina tablet (2 g/day); Placebo: starch, lactose monohydrate	BW↓; BMI↓; BFP↓; WC↓; WHR↔; TC↔; TG↓; HDL-C↔; LDL-C↔
Hernández-Lepe et al. (25)	Mexico	RCT/DB/PC Crossover	24 (100% male; SP: 12, PLA: 12)	Overweight/obese; Age: 26 ± 5; BMI: 30.9 ± 4.9	12 (6 × 6)	Spirulina capsule (4.5 g/day); Placebo: low-calorie saccharin powder	TC↓; TG↓; HDL-C↑; LDL-C↓
Ghaem Far et al. (52)	Iran	RCT/TB/PC	41 (SP: 22, PLA: 19)	HTN; Age: 50.8 ± 1.4; BMI: 29.8 ± 1.3	8	Spirulina powder (2 g/day); Placebo: chlorophyll pigment	TC↓; TG↓; HDL-C↔; LDL-C↓; FBG↔; SBP↓; DBP↓
Moradi et al. (53)	Iran	RCT/DB/PC	73 (48% male; SP: 36, PLA: 37)	UC; Age: 38.6 ± 11.3; BMI: 25.8 ± 4.7	8	Spirulina capsule (1 g/day); Placebo: inert contents	BW↔; BMI↔; WC↔; WHR↔; SBP↔; DBP↔
Koite et al. (53)	France	RCT/DB/PC	40 (55% male; SP: 20, PLA: 20)	Overweight/obese with MS; Age: 49.9 ± 9.9; BMI: 29.7 ± 2.7	12	Spirulina extract (0.02 g/day); Placebo: blue food coloring	TC↔; TG↓; HDL-C↑; LDL-C↔; FBG↔; INS↔
Rostami et al. (55)	Iran	CT	30 (37% male; SP: 15, CON: 15)	T2DM; Age: 47.0 ± 8.3; BMI: 27.7 ± 2.0	8	Spirulina tablet (4 g/day)	TC↓; TG↓; HDL-C↔; LDL-C↓; FBG↔; INS↔
Mohammad et al. (57)	Iran	CT/SB	30 (100% male; SP: 15, PLA: 15)	Overweight/obese; Age: 37.4 ± 9.9; BMI: 31.7 ± 3.9	8	Spirulina capsule (1 g/day); Placebo: starch	FBG↔
Armannia et al. (58)	Iran	RCT/TB/PC	24 (50% male; SP: 12, PLA: 12)	Obese; Age: 44.8 ± 3.1; BMI: 40.3 ± 6.0	8	Spirulina capsule (2 g/day); Placebo: not specified	BW↔; BMI↔; WHR↔; BFP↔
Supriya et al. (59)	Iran	RCT	22 (100% male; SP: 11, PLA: 11)	Obese; Age: 25–40; BMI: 33.0 ± 1.0	12	Spirulina capsule (6 g/day); Placebo: corn starch	BW↔; BMI↔; TC↓; TG↓; HDL-C↑; LDL-C↓
Bandarrigi et al. (60)	Iran	CT	20 (0% male; SP: 10, PLA: 10)	Overweight/obese; Age: 62.9 ± 1.9; BMI: 29.2 ± 1.1	8	Spirulina capsule (1.5 g/day); Placebo: starch	BMI↔; TC↔; TG↔; HDL-C↔; LDL-C↔
Delfan et al. (61)	Iran	RCT	22 (100% male; SP: 11, PLA: 11)	Obese; Age: 25–40; BMI: 33.1 ± 1.0	12	Spirulina capsule (6 g/day); Placebo: corn starch	FBG↓; INS↓
Hossein et al. (42)	Iran	RCT/SB/PC	30 (100% male; SP: 15, PLA: 15)	Overweight/obese; Age: 37.4 ± 9.9; BMI: 31.7 ± 3.9	8	Spirulina capsule (1 g/day); Placebo: starch	BW↓; BFP↓

CT, non-randomized controlled trial; RCT, randomized controlled trial; SB, single-blind; DB, double-blind; TB, triple-blind; PC: placebo-controlled; SP, Spirulina; CON, control; PLA, placebo; T2DM, type 2 diabetes; HIV-ART-naïve, treatment-naïve HIV on antiretroviral therapy; HTN, hypertension; UC, ulcerative colitis; MS, metabolic syndrome.

**TABLE 2:** Characteristics of included studies on the effects of Spirulina supplementation combined with exercise.

Study	Country	Design	Sample size	Participant details	Duration (Weeks)	Supplementation protocol	Exercise protocol	Outcomes
Hernández-Lepe et al. (26)	Mexico	RCT/DB/PC Crossover	28 (100% male; SP+EX: 14, PLA+EX: 14)	Overweight/obese; Age: 26 ± 5; BMI: 29.7 ± 2.9	12 (6 × 6)	Spirulina capsule (4.5 g/day); Placebo: low-calorie saccharin powder	RE+AE (5 sessions/week)	BMI↔; BFP↔; VO2max↔
Hernández-Lepe et al. (25)	Mexico	RCT/DB/PC Crossover	28 (100% male; SP+EX: 14, PLA+EX: 14)	Overweight/obese; Age: 26 ± 5; BMI: 29.7 ± 2.9	12 (6 × 6)	Spirulina capsule (4.5 g/day); Placebo: low-calorie saccharin powder	RE+AE (5 sessions/week)	TC↓; TG↓; HDL-C↑; LDL-C↓
Golestani et al. (27)	Iran	RCT/SB/PC	20 (0% male; SP+EX: 10, PLA+EX: 10)	Overweight/obese; Age: 21.6 ± 1.8; BMI: 29.3 ± 3.0	4	Spirulina pill (1 g/day); Placebo: starch	HIIT (3 sessions/week)	BW↔; BMI↔; BFP↔; TC↔; TG↔; HDL-C↔; LDL-C↔
Nobari et al. (56)	Iran	CT/DB	20 (0% male; SP+EX: 10, PLA+EX: 10)	Overweight/obese; Age: 25.0 ± 7.0; BMI: 28.2 ± 3.3	8	Spirulina powder (6 g/day); Placebo: green food coloring	HIIT (3 sessions/week)	BW↔; BMI↔; BFP↔; VO2max↔
Supriya et al. (59)	Iran	RCT	22 (100% male; SP+EX: 11, PLA+EX: 11)	Obese; Age: 25–40; BMI: 33.0 ± 0.9	12	Spirulina capsule (6 g/day); Placebo: corn starch	HIIT (3 sessions/week)	BW↔; BMI↔; TC↔; TG↔; HDL-C↑; LDL-C↓
Bandarrigi et al. (60)	Iran	CT	20 (0% male; SP+EX: 10, PLA+EX: 10)	Overweight/obese; Age: 61.8 ± 2.7; BMI: 29.5 ± 1.1	8	Spirulina capsule (1.5 g/day); Placebo: starch	WYP (2–3 sessions/week)	BMI↔; TC↓; TG↓; HDL-C↑; LDL-C↓
Hossein et al. (42)	Iran	RCT/SB/PC	30 (100% male; SP+EX: 15, PLA+EX: 15)	Overweight/obese; Age: 36.5 ± 6.2; BMI: 32.0 ± 4.3	8	Spirulina capsule (1 g/day); Placebo: starch	CRT (3 sessions/week)	BW↔; BFP↔

SP+EX, Spirulina + exercise; PLA+EX, placebo + exercise; RE+AE, resistance and aerobic exercise; HIIT, high-intensity interval training; WYP, weight-bearing yoga practice; CRT, circuit resistance training.

### 3.3 Effects of spirulina supplementation alone

**FIGURE 2:**
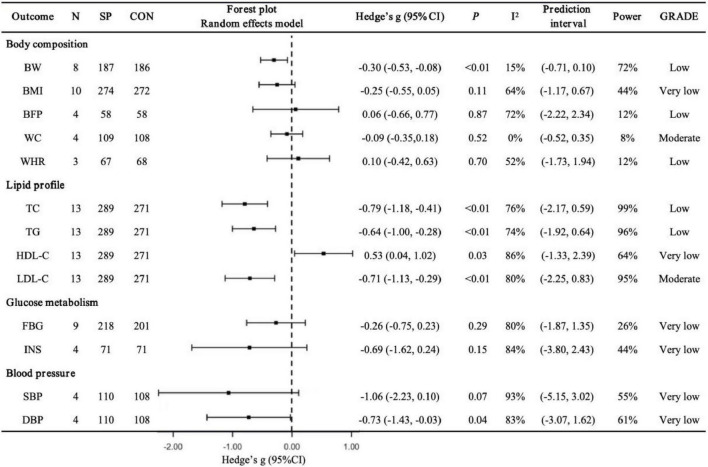
Pooled effects of Spirulina vs. control on cardiometabolic health outcomes. N, number of trials; SP, Spirulina; CON, placebo or control; Hedges’ g, effect size; 95% CI, confidence interval; p, *p*-value; I^2^, heterogeneity; Power, statistical power; GRADE, Grading of Recommendations Assessment, Development, and Evaluation; BW, body weight; BMI, body mass index; BFP, body fat percentage; WC, waist circumference; WHR, waist-to-hip ratio; TC, total cholesterol; TG, triglycerides; HDL-C, high-density lipoprotein cholesterol; LDL-C, low-density lipoprotein cholesterol; FBG, fasting blood glucose; INS, insulin; SBP: systolic blood pressure; DBP, diastolic blood pressure

Body composition (Figure 2). Spirulina significantly reduced BW compared to controls (*N* = 8, *g* = −0.30, 95% CI: −0.53 to −0.08, *I*^2^ = 15%, *p* < 0.01, low GRADE). No significant effects were observed for BMI (*N* = 10, *g* = −0.25, 95% CI: −0.55 to 0.05, *I*^2^ = 64%, *p* = 0.11, very low GRADE), BFP (*N* = 4, *g* = 0.06, 95% CI: −0.66 to 0.77, *I*^2^ = 72%, *p* = 0.87, low GRADE), WC (*N* = 4, *g* = −0.09, 95% CI: −0.35 to 0.18, *I*^2^ = 0%, *p* = 0.52, moderate GRADE), or WHR (*N* = 3, *g* = 0.10, 95% CI: −0.42 to 0.63, *I*^2^ = 52%, *p* = 0.70, low GRADE). Sensitivity analyses showed that excluding Miczke et al. (47) altered the BW pooled result (*g* = −0.17, 95% CI: −0.40 to 0.06, *p* = 0.14), and excluding Ngo-Matip et al. (45) altered the BMI pooled result (*g* = −0.35, 95% CI: −0.62 to −0.08, *p* = 0.01), though the overall direction of findings remained consistent.

Lipid profile (Figure 2). Spirulina significantly lowered TC (*N* = 13, *g* = −0.79, 95% CI: −1.18 to −0.41, *I*^2^ = 76%, *p* < 0.01, low GRADE), TG (*N* = 13, *g* = −0.64, 95% CI: −1.00 to −0.28, *I*^2^ = 74%, *p* < 0.01, low GRADE), and LDL-C (*N* = 13, *g* = −0.71, 95% CI: −1.13 to −0.29, *I*^2^ = 80%, *p* < 0.01, moderate GRADE), while increasing HDL-C (*N* = 13, *g* = 0.53, 95% CI: 0.04 to 1.02, *I*^2^ = 86%, *p* = 0.03, very low GRADE). Sensitivity analyses indicated that excluding Mani et al. (43) (*g* = 0.48, 95% CI: −0.03 to 0.99, *p* = 0.07), Ghaem Far et al. (52) (*g* = 0.45, 95% CI: −0.06 to 0.95, *p* = 0.08), or Supriya et al. (59) (*g* = 0.50, 95% CI: −0.02 to 1.02, *p* = 0.06) altered the HDL-C pooled result, but the overall direction remained consistent.

Glucose metabolism (Figure 2). Spirulina showed no significant effect on FBG (*N* = 9, *g* = −0.26, 95% CI: −0.75 to 0.23, *I*^2^ = 80%, *p* = 0.29, very low GRADE) or INS (*N* = 4, *g* = −0.69, 95% CI: −1.62 to 0.24, *I*^2^ = 84%, *p* = 0.15, very low GRADE). Sensitivity analyses confirmed that excluding any single study did not alter the overall findings.

Blood pressure (Figure 2). Spirulina significantly reduced DBP (*N* = 4, *g* = −0.73, 95% CI: −1.43 to −0.03, *I*^2^ = 83%, *p* = 0.04, very low GRADE) and showed a trend toward reducing SBP (*N* = 4, *g* = −1.06, 95% CI: −2.23 to 0.10, *I*^2^ = 93%, *p* = 0.07, very low GRADE). Sensitivity analyses revealed that excluding Jensen et al. (46) altered the SBP pooled result (*g* = −1.47, 95% CI: −2.89 to −0.05, *p* = 0.04), and excluding Jensen et al. (46) (*g* = −0.85, 95% CI: −1.73 to 0.03, *p* = 0.06), Miczke et al. (47) (*g* = −0.61, 95% CI: −1.55 to 0.34, *p* = 0.21), or Ghaem Far et al. (52) (*g* = −0.48, 95% CI: −1.20 to 0.25, *p* = 0.20) altered the DBP pooled result, though the overall direction remained consistent.

Forest plots and sensitivity analyses for all outcomes are presented in Supplementary materials 1–4, 7–10.

### 3.4 Effects of spirulina combined with exercise

**FIGURE 3:**
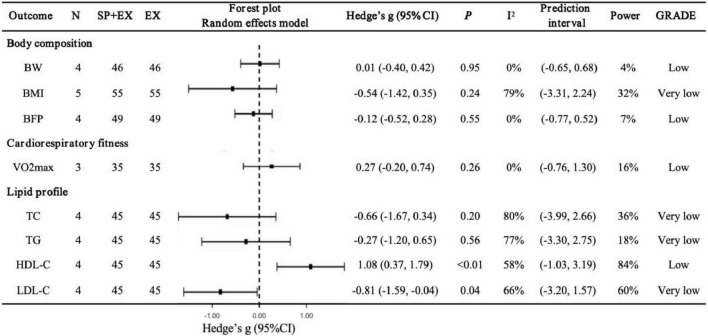
Pooled effects of Spirulina + exercise vs. exercise alone on cardiometabolic health outcomes. SP+EX, Spirulina + exercise; EX, exercise alone; VO2max, maximal oxygen uptake.

Body composition and cardiorespiratory fitness (Figure 3). Compared to placebo + exercise, Spirulina combined with exercise showed no significant effects on BW (*N* = 4, *g* = 0.01, 95% CI: −0.40 to 0.42, *I*^2^ = 0%, *p* = 0.95, low GRADE), BMI (*N* = 5, *g* = −0.54, 95% CI: −1.42 to 0.35, *I*^2^ = 79%, *p* = 0.24, very low GRADE), BFP (*N* = 4, *g* = −0.12, 95% CI: −0.52 to 0.28, *I*^2^ = 0%, *p* = 0.55, low GRADE), or VO2max (*N* = 3, *g* = 0.27, 95% CI: −0.20 to 0.74, *I*^2^ = 0%, *p* = 0.26, low GRADE). Sensitivity analyses confirmed that excluding any single study did not alter the overall findings.

Lipid profile (Figure 3). Spirulina combined with exercise significantly improved HDL-C (*N* = 4, *g* = 1.08, 95% CI: 0.37–1.79, *I*^2^ = 58%, *p* < 0.01, low GRADE) and LDL-C (*N* = 4, *g* = −0.81, 95% CI: −1.59 to −0.04, *I*^2^ = 66%, *p* = 0.04, very low GRADE). No significant effects were observed for TC (*N* = 4, *g* = −0.66, 95% CI: −1.67 to 0.34, *I*^2^ = 80%, *p* = 0.20, very low GRADE) or TG (*N* = 4, *g* = −0.27, 95% CI: −1.20 to 0.65, *I*^2^ = 77%, *p* = 0.56, very low GRADE). Sensitivity analyses showed that excluding Hernández-Lepe et al. (25) (g = −0.95, 95% CI: −2.08 to 0.19, *p* = 0.10), Supriya et al. (59) (*g* = −0.79, 95% CI: −1.86 to 0.29, *p* = 0.15), or Bandarrigi et al. (60) (*g* = −0.48, 95% CI: −1.03 to 0.06, *p* = 0.08) altered the LDL-C pooled result, though the overall direction remained consistent.

Forest plots and sensitivity analyses for all outcomes are presented in Supplementary materials 5–6, 11–12.

### 3.5 Moderator analysis

Subgroup analyses (Supplementary materials 13–16) revealed that participant age, BMI, health status, Spirulina form, dose, and intervention duration moderated the effects of Spirulina supplementation on lipid profiles. Notably, greater improvements were observed in participants with type 2 diabetes (T2DM; TC: *g* = −1.26; TG: *g* = −1.22; HDL-C: *g* = 0.67; LDL-C: *g* = −1.04) and hypertension (HTN; TC: *g* = −1.18; LDL-C: *g* = −1.40). Powdered Spirulina (TC: *g* = −1.61; TG: *g* = −1.41; HDL-C: *g* = 1.80; LDL-C: *g* = −1.62) outperformed tablets or capsules. TC improvements were more pronounced in participants aged 45–59 years (*g* = −0.98) and at Spirulina doses of 4–10 g/day (*g* = −1.08). TG improvements were greater at baseline BMI of 25–30 kg/m^2^ (*g* = −0.98), while LDL-C improvements were optimal at 2 g/day.

**FIGURE 4:**
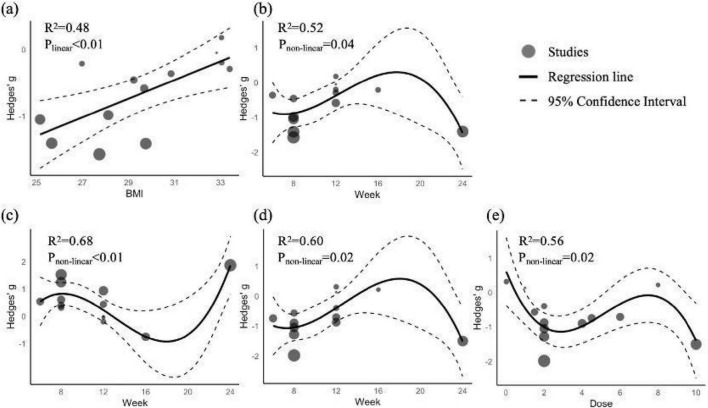
Regression plots of TG, HDL-C, and LDL-C as functions of BMI, Spirulina dose, and intervention duration. **(a)** TG vs. BMI; **(b)** TG vs. intervention duration; **(c)** HDL-C vs. intervention duration; **(d)** DL-C vs. intervention duration; **(e)** LDL-C vs. Spirulina dose.

Regression analyses (Figure 4) identified relationships between BMI, Spirulina dose, intervention duration, and post-supplementation TG, HDL-C, and LDL-C levels, with no moderating effects observed for other variables (Supplementary materials 17–18). BMI exhibited a linear relationship with TG (*R*^2^ = 0.48, *p* < 0.01; Figure 4a), with lower BMI linked to greater TG reductions. Intervention duration showed a non-linear relationship with TG (*R*^2^ = 0.52, *p* = 0.04; Figure 4b), HDL-C (*R*^2^ = 0.68, *p* < 0.01; Figure 4c), and LDL-C (*R*^2^ = 0.56, *p* = 0.02; Figure 4d), with optimal effects at 7–8 weeks (TG: *g* = −0.91; HDL-C: *g* = 0.83; LDL-C: *g* = −1.07) and 24 weeks (TG: *g* = −1.44; HDL-C: *g* = 1.87; LDL-C: *g* = −1.53). Spirulina dose had a non-linear relationship with LDL-C (*R*^2^ = 0.60, *p* = 0.02; Figure 4e), with peak effects at 2.8 g (*g* = −1.14) and 10 g (*g* = −1.40).

### 3.6 Risk of bias

Using the Cochrane Risk of Bias 2.0 assessment, 10 studies (25, 26, 47–53, 58) were rated as low risk, 11 studies (42, 44–46, 52, 54, 56, 57, 59–61) had some concerns, and 2 studies (43, 55) were high risk. Randomization process issues led to high-risk ratings for two studies (43, 55) due to non-random allocation sequences and some concerns for seven studies (44–46, 56, 59–61) due to inadequate allocation concealment. Deviations from intended interventions caused concerns in 10 studies (27, 42–45, 55, 57, 59–61) due to blinding limitations. Incomplete outcome reporting raised concerns in two studies (54, 57). A detailed risk of bias summary, including per-study scores, is provided in Supplementary material 19.

### 3.7 Publication bias

Publication bias for Spirulina’s effects on BMI and lipid profiles was evaluated using funnel plots and Egger’s test (Supplementary Material 20). No significant bias was detected for BMI (*p* = 0.31), TC (*p* = 0.32), TG (*p* = 0.36), HDL-C (*p* = 0.18), or LDL-C (*p* = 0.47).

## 4 Discussion

This meta-analysis comprehensively evaluated the effects of Spirulina supplementation, alone or combined with exercise, on cardiometabolic health in overweight and obese adults, incorporating 23 studies (1,035 participants, BMI > 25 kg/m^2^). Spirulina alone significantly improved BW, TC, TG, HDL-C, LDL-C, and DBP, but had no notable impact on BMI, BFP, WC, WHR, FBG, INS, SBP. When combined with exercise, Spirulina further enhanced improvements in HDL-C and LDL-C but provided no additional benefits for TC, TG, BW, BMI, BFP, or VO2max. Subgroup analyses revealed that powdered Spirulina was more effective in improving lipid profiles in participants with T2DM or HTN. Individuals aged 45–59 years receiving 4–10 g/day exhibited greater TC improvements. Regression analyses indicated that lower BMI was associated with larger TG reductions, doses of 2.8 or 10 g optimized LDL-C reductions, and intervention durations of 7–8 weeks or 24 weeks maximized improvements in TG, HDL-C, and LDL-C.

### 4.1 Effects of spirulina supplementation alone

#### 4.1.1 Lipid profile

Our findings demonstrate that Spirulina significantly improved TC (*g* = −0.79), TG (*g* = −0.64), HDL-C (*g* = 0.53), and LDL-C (*g* = −0.71), consistent with a recent systematic review and meta-analysis (21) reporting Spirulina’s lipid-modulating effects. These improvements underscore Spirulina’s role in lipid metabolism, likely due to its bioactive compounds. Phycocyanin and phycocyanobilin in Spirulina inhibit NADPH oxidase, reducing pro-inflammatory cytokines (e.g., interleukin-6, tumor necrosis factor-α) and enhancing antioxidant enzyme activity (e.g., superoxide dismutase, glutathione), thereby mitigating oxidative stress, lipid peroxidation, and dyslipidemia-related inflammation (12, 62). Glycolipid H-b2 and phycocyanin suppress pancreatic lipase activity, reducing intestinal fat digestion and absorption (63). Additionally, γ-linolenic acid and niacin in Spirulina improve dyslipidemia (12, 21), while Spirulina inhibits jejunal cholesterol absorption and ileal bile acid reabsorption (64).

Subgroup analyses revealed greater improvements in participants with T2DM (TC: *g* = −1.26; TG: *g* = −1.22; HDL-C: *g* = 0.67; LDL-C: *g* = −1.04) and HTN (TC: *g* = −1.18; LDL-C: *g* = −1.40), possibly due to heightened sensitivity in individuals with baseline metabolic dysfunction. Hatami et al. (18) corroborated these findings, reporting significant lipid improvements in T2DM patients. Spirulina’s form also influenced efficacy, with powder (TC: *g* = −1.61; TG: *g* = −1.41; HDL-C: *g* = 1.80; LDL-C: *g* = −1.62) outperforming tablets or capsules, likely due to higher nutrient content (65) and enhanced digestibility and bioavailability from the absence of a cellulose cell wall (66). Dose subgroup analyses indicated that 2 g/day optimized LDL-C improvement (*g* = −1.08), while 4–10 g/day was more effective for TC (*g* = −1.08), suggesting dose-dependent effects. Regression analyses revealed a non-linear dose-response for LDL-C, with optimal effects at 2.8 g (*g* = −1.14) and 10 g (*g* = −1.40), and a non-linear relationship between intervention duration and lipids, with short-term benefits at 7–8 weeks (TG: *g* = −0.91; HDL-C: *g* = 0.83; LDL-C: *g* = −1.07) and long-term effects at 24 weeks (TG: *g* = −1.44; HDL-C: *g* = 1.87; LDL-C: *g* = −1.53), possibly reflecting short-term metabolic adaptations and long-term nutritional changes. However, the limited number of studies with 12–24 week interventions renders long-term effects uncertain, necessitating further high-quality research. A linear relationship between BMI and TG indicated greater TG improvement at lower BMI, suggesting Spirulina is more effective in overweight than severely obese individuals. These findings are significant for cardiovascular health, as elevated TG and LDL-C are major risk factors for atherosclerosis, coronary heart disease, and stroke (67–69).

#### 4.1.2 Body composition

Spirulina significantly reduces BW (*g* = −0.30), consistent with findings by Zarezadeh et al. (20). No significant changes were observed in BMI (*g* = −0.25), BFP (*g* = 0.06), WC (*g* = −0.09), or WHR (*g* = 0.10). This may be attributed to Spirulina’s high protein content (∼ 60–70% dry weight) and amino acid profile, which likely support muscle protein synthesis or maintenance of fat-free mass (FFM) (10, 70), potentially offsetting fat loss in BFP, WC, and WHR. Nobari et al. (56) and Delfan et al. (61) corroborate this, reporting a slight increase in FFM following Spirulina supplementation. Furthermore, Spirulina primarily regulates BW by inhibiting macrophage infiltration into visceral fat, reducing hepatic fat accumulation, alleviating oxidative stress, enhancing insulin sensitivity, and increasing satiety (10, 12), rather than through direct lipolysis or adipose tissue remodeling.

#### 4.1.3 Glucose metabolism

Spirulina did not significantly improve FBG (*g* = −0.26) or INS (*g* = −0.69), aligning with Bohórquez-Medina et al. (23). However, Hatami et al. (18) and Ghanbari et al. (19) reported significant FBG improvements, and Hamedifard et al. (24) noted enhancements in both FBG and INS. These discrepancies may reflect metabolic heterogeneity among participants, as Hatami et al. and Ghanbari et al. studied T2DM patients, while Hamedifard et al. focused on metabolic syndrome patients, suggesting greater sensitivity to Spirulina’s glucose-lowering effects in these populations. Preclinical studies indicate Spirulina reduces blood glucose in diabetic rats by stimulating insulin release from pancreatic β-cells, inhibiting dipeptidyl peptidase-IV to prolong incretin effects, and reducing intestinal carbohydrate absorption (71). However, high heterogeneity, very low-GRADE certainty, and wide confidence intervals underscore the need for high-quality studies to confirm these effects.

#### 4.1.4 Blood pressure

Spirulina significantly reduced DBP (*g* = −0.73) and showed a trend toward improving SBP (*g* = −1.06, *p* = 0.07), consistent with Machowiec et al. (22). These effects may result from phycocyanin stimulating adiponectin, enhancing endothelial nitric oxide synthase (eNOS) expression and nitric oxide (NO) production, promoting vasodilation, and reducing endothelial dysfunction markers (e.g., sVCAM-1, sE-selectin) (72, 73). Spirulina-derived angiotensin-converting enzyme (ACE) inhibitory peptides (e.g., IQP, VEP) suppress angiotensin II production, reducing vasoconstriction, while bioactive peptides (e.g., SP6) induce NO-dependent vasorelaxation via the PI3K/AKT/eNOS pathway (74, 75). However, high heterogeneity, very low-GRADE certainty, and wide confidence intervals highlight the need for larger, high-quality studies to confirm these effects.

### 4.2 Synergistic effects of spirulina combined with exercise

#### 4.2.1 Lipid profile

Spirulina combined with exercise significantly improved HDL-C (*g* = 1.08) and LDL-C (*g* = −0.81), demonstrating stronger lipid-modulating effects than Spirulina alone. This synergy likely results from Spirulina’s antioxidant and anti-inflammatory properties enhancing exercise-induced lipid metabolism, such as increased hepatic lipoprotein lipase activity (76) and reduced lipid synthesis and fat storage via AMPK signaling pathway activation and downregulation of lipid droplet-related genes (e.g., Plin2, Rab18) (77). However, no significant changes were observed in TC (*g* = −0.66) or TG (*g* = −0.27), possibly due to participant characteristics or Spirulina supplementation protocols. Regression analysis (Figure 4) indicated that TG improvement was moderated by BMI, with higher BMI (29–33 kg/m^2^ in included studies) associated with smaller TG reductions, suggesting that severe obesity may limit the combined intervention’s efficacy. The optimal intervention window for TG was 7–8 weeks, but the varied durations (4–12 weeks) of included studies may have missed this window. Subgroup analyses (Supplementary material 13, 14) suggested that higher Spirulina doses (4–10 g/day) and powdered form may yield better TC and TG outcomes.

#### 4.2.2 Body composition and cardiorespiratory fitness

No significant changes were observed in BW (*g* = 0.01), BMI (*g* = −0.54), BFP (*g* = −0.12), or VO2max (*g* = 0.27), likely due to short intervention durations or heterogeneity in exercise protocols. Intervention durations (4–12 weeks) may have been insufficient to elicit measurable changes in BW, BMI, or BFP. Additionally, the diversity of exercise types (high-intensity interval training, resistance training, aerobic exercise, circuit training, and yoga) may have led to inconsistent effects on body composition. For VO2max, prior studies (78, 79) suggested Spirulina enhances oxygen uptake and endurance, but no significant effects were observed here, possibly because exercise protocols were not optimized for cardiopulmonary adaptations or required longer durations to detect Spirulina’s impact. These findings indicate that short-term interventions may be inadequate for significant body composition changes, and VO2max improvements may require higher-intensity or prolonged exercise stimuli. Overall, Spirulina combined with exercise shows potential to improve specific lipid markers, but optimizing exercise type and intervention duration is essential for broader metabolic benefits.

### 4.3 Clinical significance and cost-effectiveness

Our findings indicate that Spirulina significantly reduces BW (*g* = −0.30, ∼2.36 kg), representing approximately 2–3% of initial BW. While this reduction may not meet the 5–10% threshold required for substantial cardiovascular risk reduction in obese patients (80), modest weight loss may still confer meaningful health benefits. A systematic review (81) suggests that weight loss below 5% can still improve cardiovascular, metabolic, and quality-of-life outcomes, indicating Spirulina’s potential clinical relevance for overweight and obese adults, particularly as an adjunct to lifestyle interventions. However, for individuals with severe obesity, additional interventions such as energy restriction or exercise may be necessary to achieve greater weight loss. Spirulina significantly improves lipid profiles, reducing TC (*g* = −0.79, ∼18.24 mg/dL), TG (*g* = −0.64, ∼23.50 mg/dL), and LDL-C (*g* = −0.71, ∼12.44 mg/dL), while increasing HDL-C (*g* = 0.53, ∼4.20 mg/dL). These changes are clinically significant. Elevated TC levels are positively associated with non-hemorrhagic stroke and total cardiovascular mortality (82). A 10 mg/dL reduction in TG is linked to a 1.6 or 1.4% decrease in mortality, myocardial infarction, and recurrent acute coronary syndrome (83), corresponding to a 3.8 or 3.3% reduction in this study. Each 1 mg/dL increase in HDL-C reduces coronary artery disease risk by 2% in men and 3% in women (84), corresponding to an 8.4% and 12.6% risk reduction for men and women, respectively, in this study. A 10 mg/dL reduction in LDL-C lowers the relative risk of coronary heart disease mortality by 7.2% and coronary events by 7.1% (85), corresponding to reductions of 9 and 8.8% in this study. These findings support Spirulina as a non-pharmacological or adjunctive strategy for dyslipidemia patients at increased cardiovascular risk. Indeed, research (86) demonstrates that Spirulina, as an adjunct to metformin, outperforms metformin alone in long-term blood glucose and lipid control in T2DM patients, without significant adverse effects or hepatic/renal complications. Spirulina significantly reduces DBP (*g* = −0.73, ∼2.60 mmHg). Clinically, a 2 mmHg reduction in DBP decreases hypertension prevalence by 17%, coronary heart disease risk by 6%, and stroke/transient ischemic attack risk by 15% (87). Thus, our results endorse Spirulina supplementation as a clinically significant non-pharmacological strategy for blood pressure control, though high heterogeneity limits certainty.

The cost-effectiveness of Spirulina supplementation is a key consideration. Commercially available Spirulina is relatively inexpensive (2–3 g daily costs ∼$0.5–2, depending on brand and region) and widely used, with no serious adverse events reported in included studies. Compared to pharmacological interventions (e.g., statins or antihypertensives), Spirulina offers a lower-cost, minimal-side-effect alternative, making it a potentially cost-effective adjunctive therapy, particularly in resource-limited settings. However, it may be insufficient as a standalone treatment for severe metabolic disorders, suggesting its optimal role within comprehensive intervention strategies, including exercise and dietary modifications. Long-term studies are needed to evaluate the sustained cost-effectiveness of Spirulina supplementation.

### 4.4 Future research directions

Future studies should prioritize large-scale, high-quality randomized controlled trials with standardized designs, rigorous implementation, and transparent reporting of randomization, blinding, and raw data to minimize bias. Longer interventions (≥ 12 weeks) are needed to establish the long-term efficacy and safety of Spirulina, alone or combined with exercise. Further research should investigate Spirulina’s effects in specific metabolic disease populations (e.g., T2DM, HTN) to confirm targeted benefits. Optimized exercise protocols should be developed to systematically evaluate Spirulina’s potential to enhance V02max and aerobic capacity. The interactions between Spirulina and exercise-induced metabolic pathways warrant deeper exploration to elucidate the molecular and physiological mechanisms underlying their synergy. Additionally, combining Spirulina with other interventions, such as energy restriction or pharmacotherapy, should be explored to develop comprehensive strategies for managing cardiometabolic health.

### 4.5 Strengths and limitations

This study comprehensively evaluated Spirulina’s effects on multiple cardiometabolic health markers, including body composition, lipid profiles, glucose metabolism, and blood pressure, providing a holistic view of its benefits in overweight and obese adults. It is the first to systematically review the synergistic effects of Spirulina combined with exercise. Subgroup and regression analyses elucidated how participant characteristics (age, BMI, health status) and intervention protocols (form, dose, duration) influence outcomes, offering refined guidance for Spirulina interventions. However, the exclusion of studies lacking key data (e.g., BMI) may have omitted relevant findings. High heterogeneity (*I*^2^ > 75%) and very low-GRADE certainty for outcomes like glucose metabolism and blood pressure reduce confidence in pooled results, likely due to variability in participant characteristics, Spirulina protocols, or study designs. Most interventions lasted ≤ 12 weeks, limiting insights into Spirulina’s sustained effects. Only seven studies examined Spirulina with exercise, and the diversity of exercise modalities (high-intensity interval training, yoga, resistance training) hindered robust conclusions about synergistic effects.

## 5 Conclusion and recommendations

Spirulina supplementation alone significantly improves BW, lipid profiles, and blood pressure in overweight and obese adults, particularly those with metabolic disorders such as T2DM or HTN. Combining Spirulina with exercise further enhances specific lipid outcomes (HDL-C and LDL-C). These findings suggest that Spirulina is a valuable adjunct for managing cardiometabolic risk factors, especially in individuals with dyslipidemia or hypertension.

For overweight or obese individuals with metabolic disorders, a daily dose of 2–3 g of powdered Spirulina for 7–8 weeks is recommended to optimize lipid profiles, with longer-term supplementation (24 weeks) potentially sustaining these benefits. Combining Spirulina with exercise can amplify lipid improvements, indicating that integrating Spirulina with lifestyle interventions may yield superior metabolic outcomes. However, for severely obese individuals, additional weight-loss interventions are necessary, as Spirulina’s effects on TG and body composition are limited in this population.

## Data Availability

The original contributions presented in this study are included in this article/Supplementary material, further inquiries can be directed to the corresponding author.
