# Supplementary material for: Effects of spirulina supplementation alone or with exercise on cardiometabolic health in overweight and obese adults: a systematic review and meta-analysis
Source: Front Nutr. 2025 Jun 27;12:1624982. doi: 10.3389/fnut.2025.1624982 (PMC12245695; doi:10.3389/fnut.2025.1624982)

**Forest plot: Supplementary material 1-6**

**Sensitivity analyses: Supplementary materials 7-12**

**Subgroup analyses: Supplementary materials 13-16**

**Regression analyses: Supplementary material 17-18**

**Risk of bias assessment: Supplementary material 19**

**Funnel plots: Supplementary material 20**

**Supplementary material 1**: Forest plot of Spirulina versus control effects on body composition


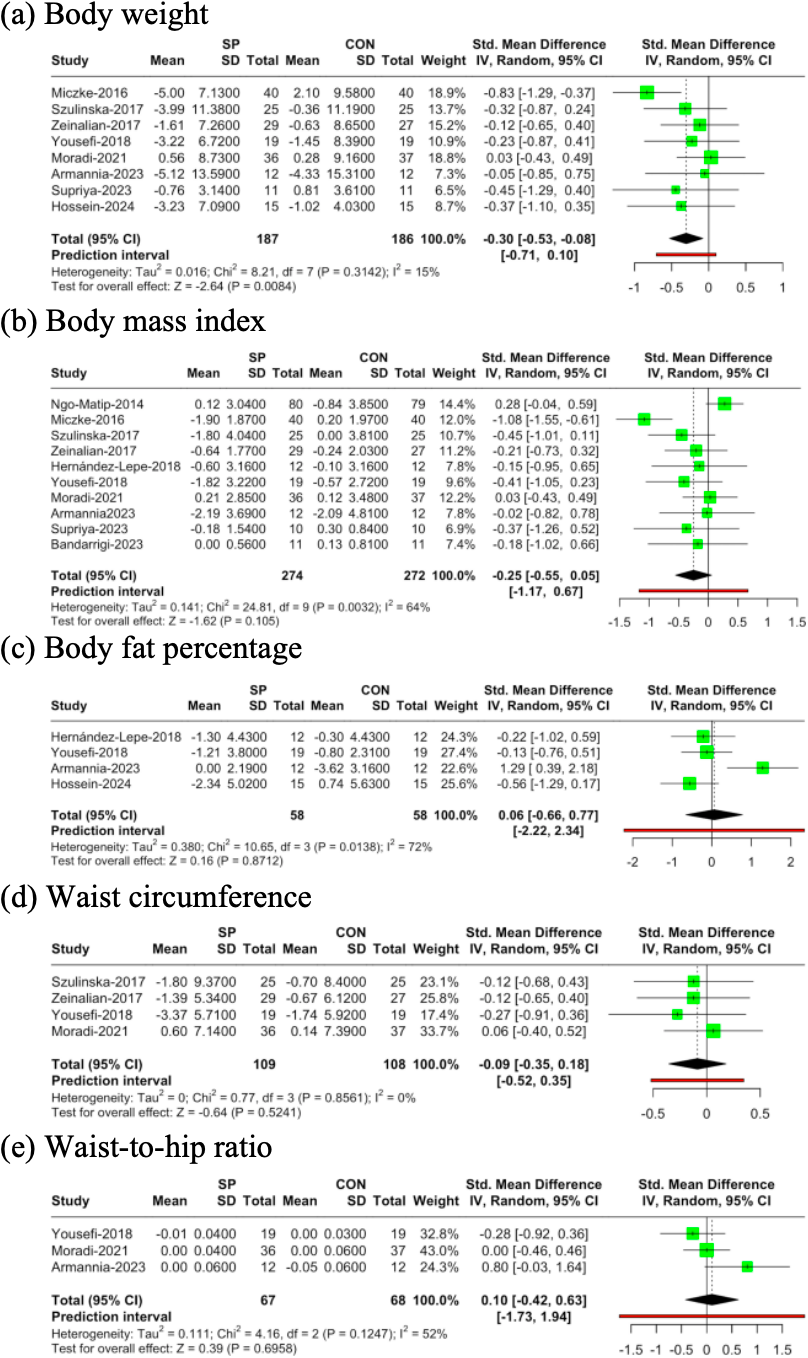


**Supplementary material 2**: Forest plot of Spirulina versus control effects on lipid profiles


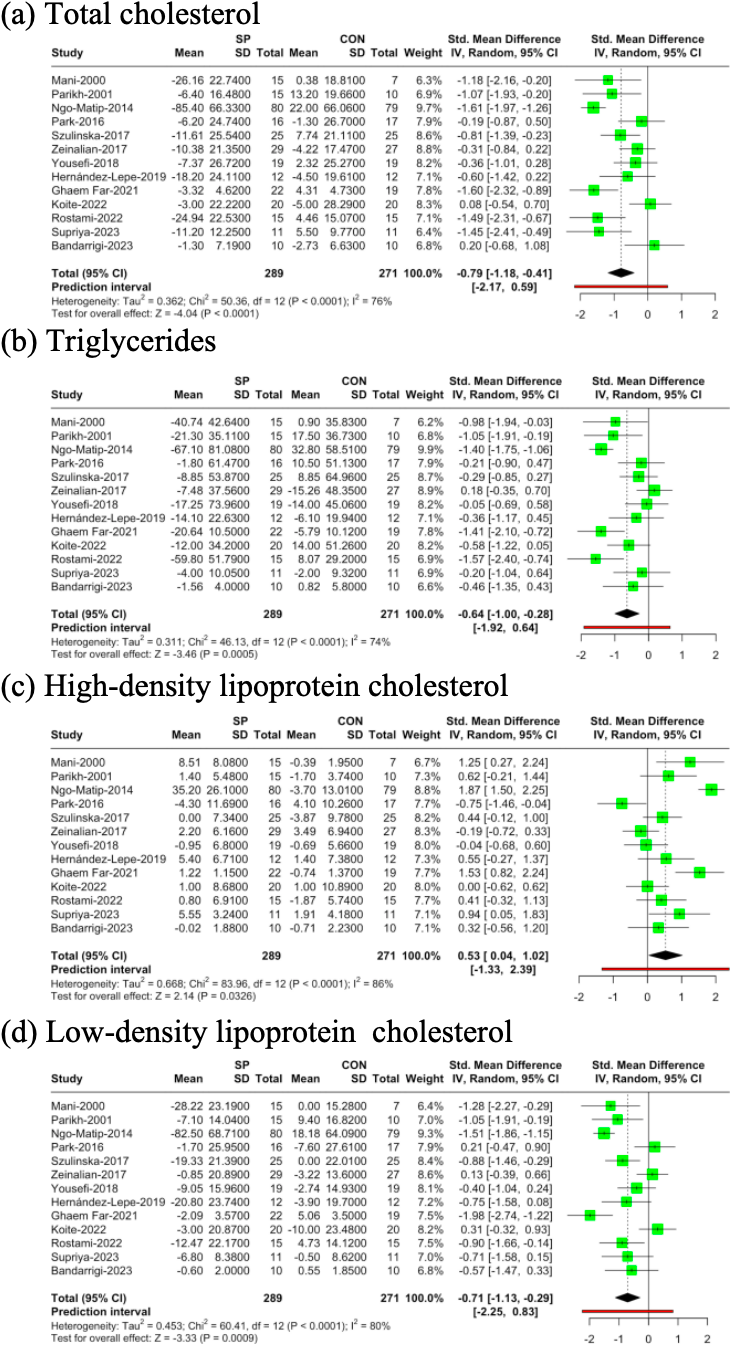


**Supplementary material 3**: Forest plot of Spirulina versus control effects on glucose metabolism


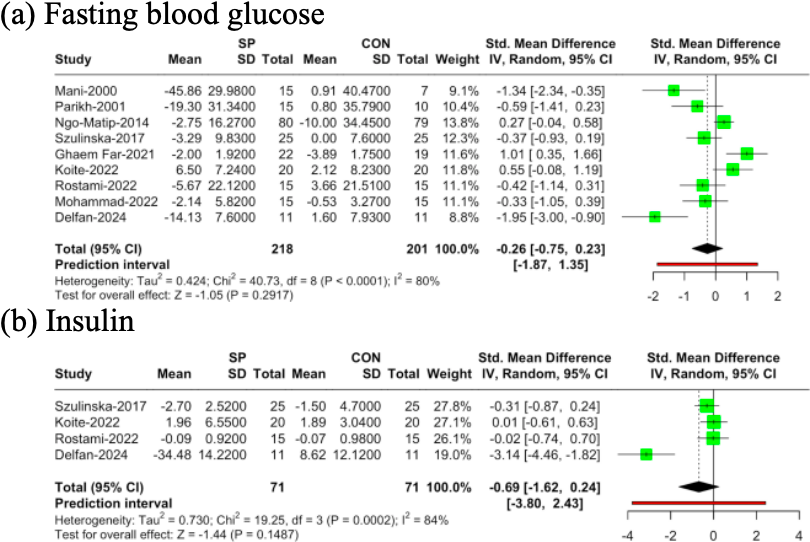


**Supplementary material 4**: Forest plot of Spirulina versus control effects on blood pressure


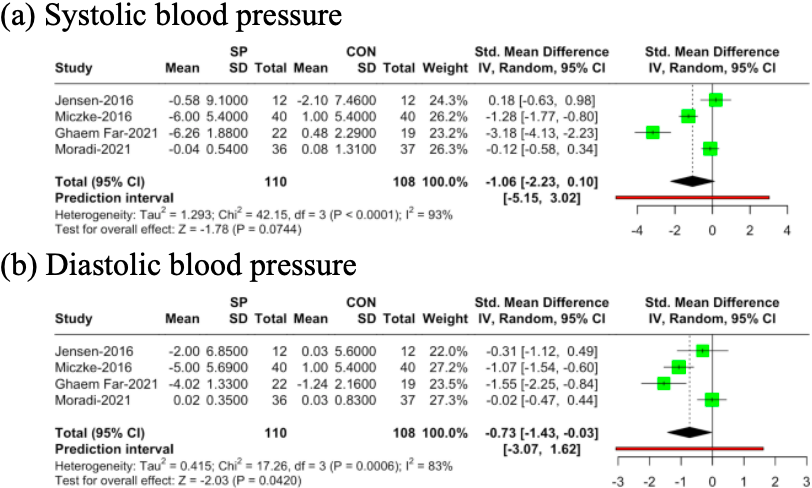


**Supplementary material 5**: Forest plot of Spirulina + exercise versus exercise alone effects on body composition and cardiorespiratory fitness


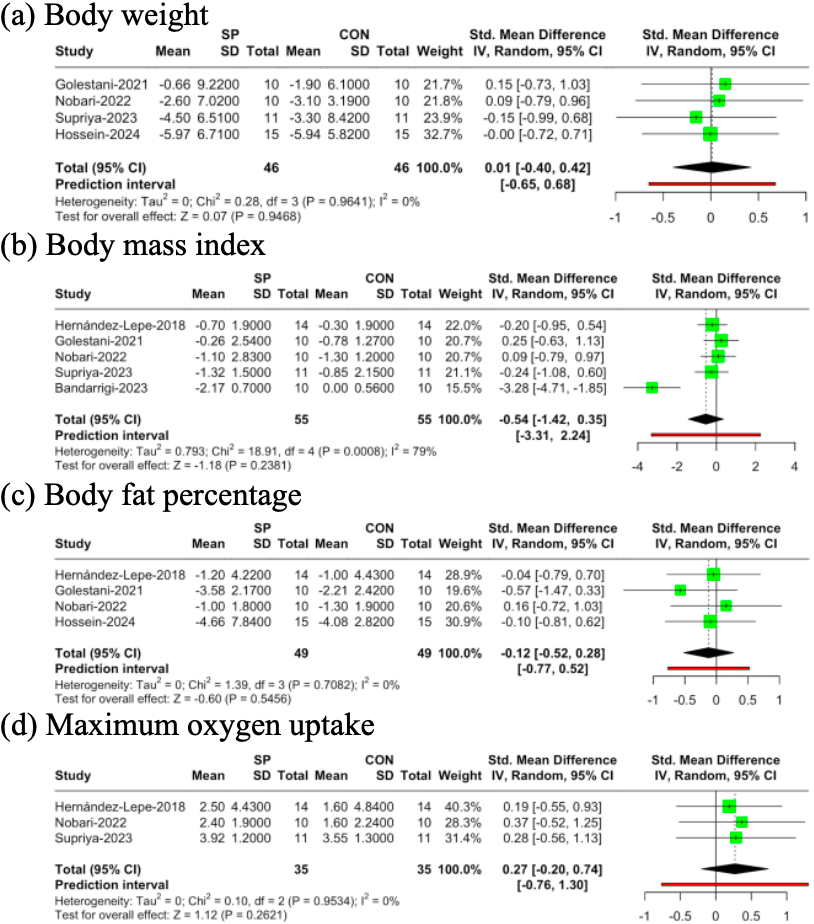


**Supplementary material 6**: Forest plot of Spirulina + exercise versus exercise alone effects on lipid profiles


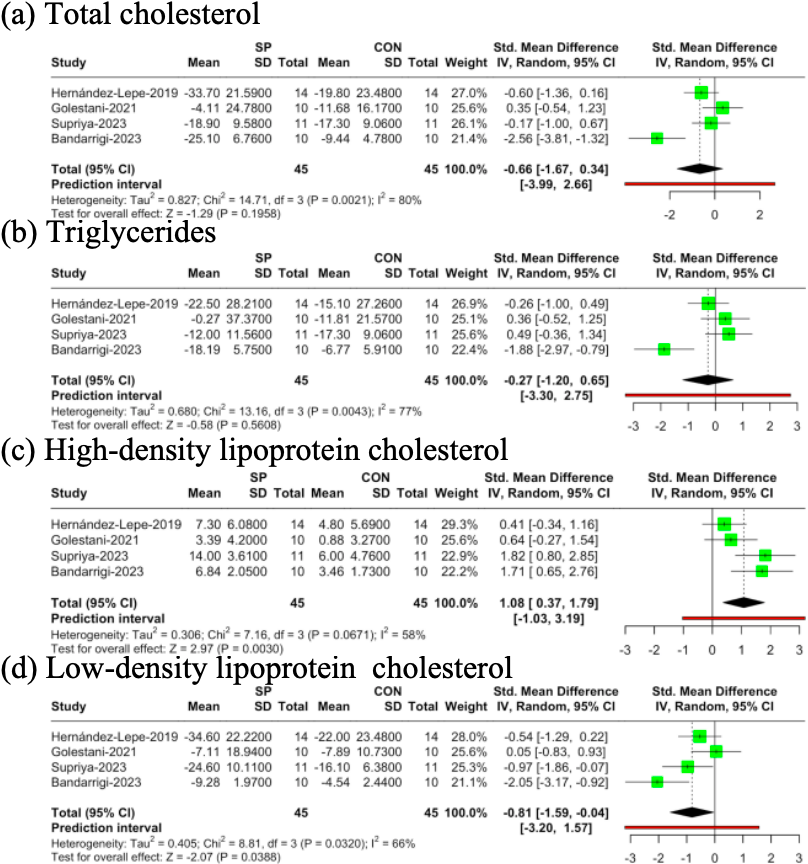


**Supplementary material 7**: Sensitivity analysis of Spirulina versus control effects on body composition


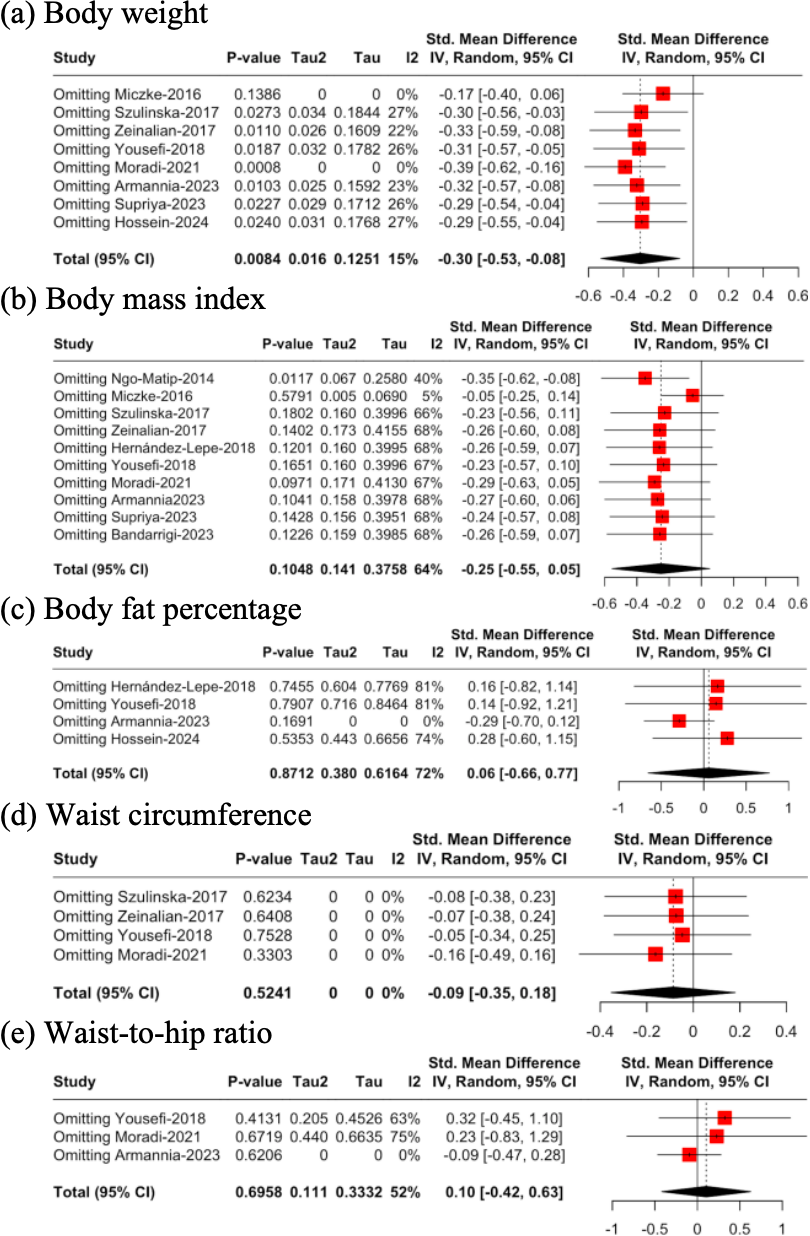


**Supplementary material 8**: Sensitivity analysis of Spirulina versus control effects on blood lipids


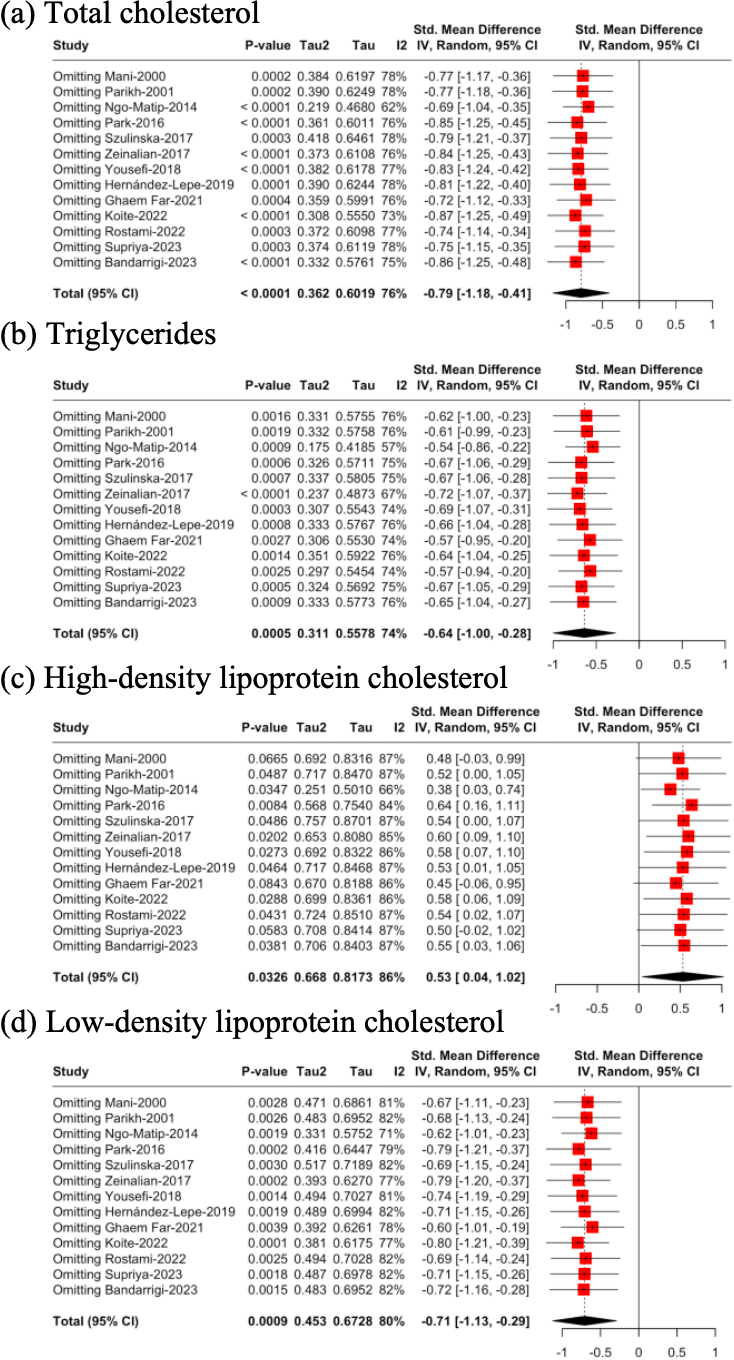


**Supplementary material 9**: Sensitivity analysis of Spirulina versus control effects on glucose metabolism


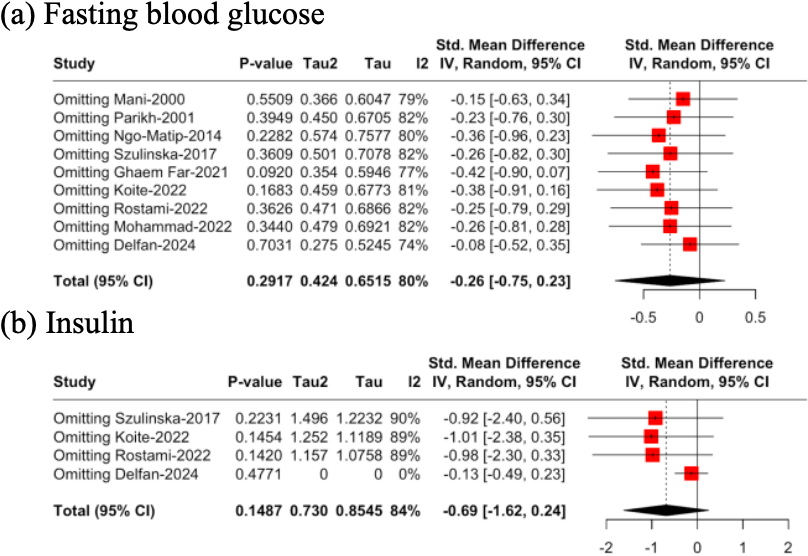


**Supplementary material 10**: Sensitivity analysis of Spirulina versus control effects on blood pressure


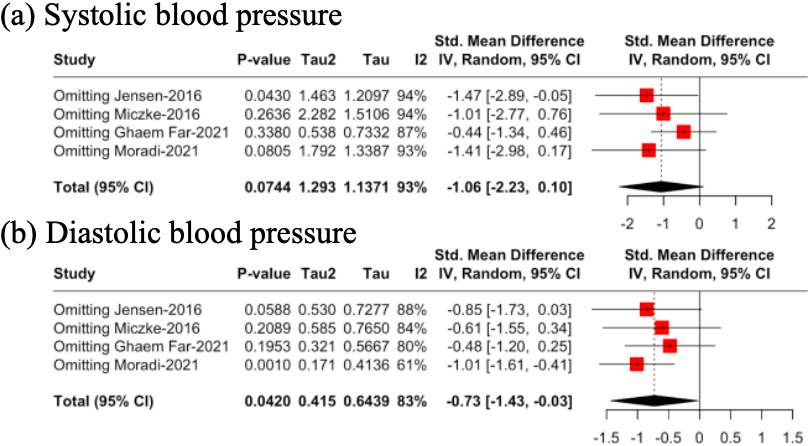


**Supplementary material 11**: Sensitivity analysis of Spirulina + exercise versus exercise alone effects on body composition and cardiorespiratory fitness


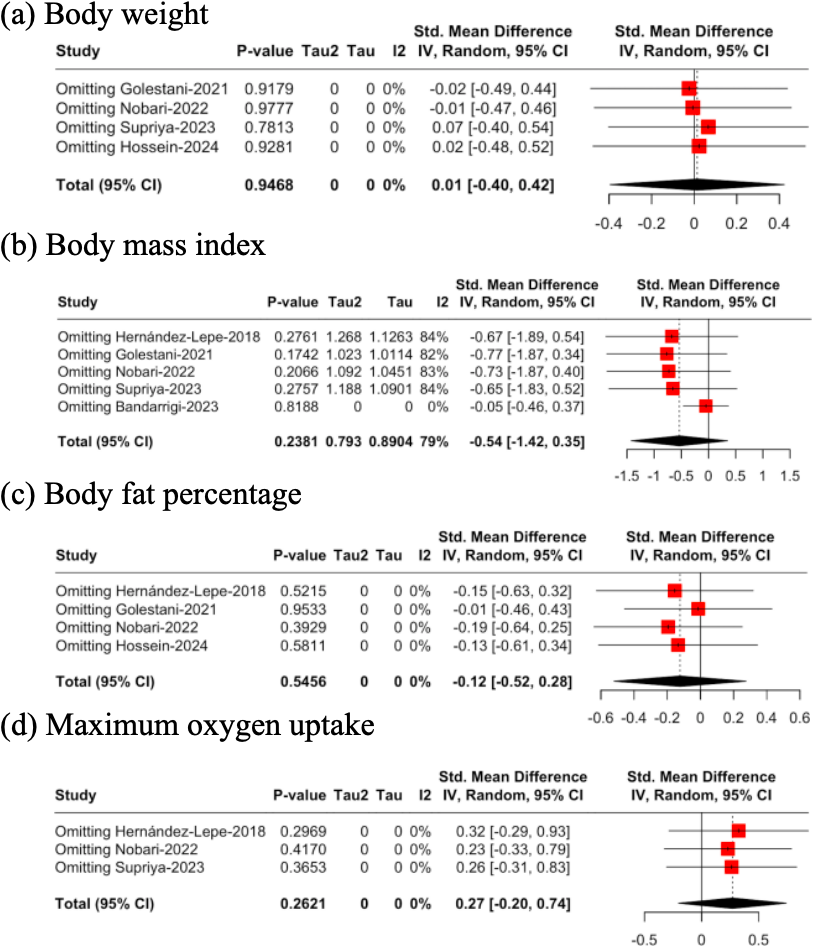


**Supplementary material 12**: Sensitivity analysis of Spirulina + exercise versus exercise alone effects on blood lipids


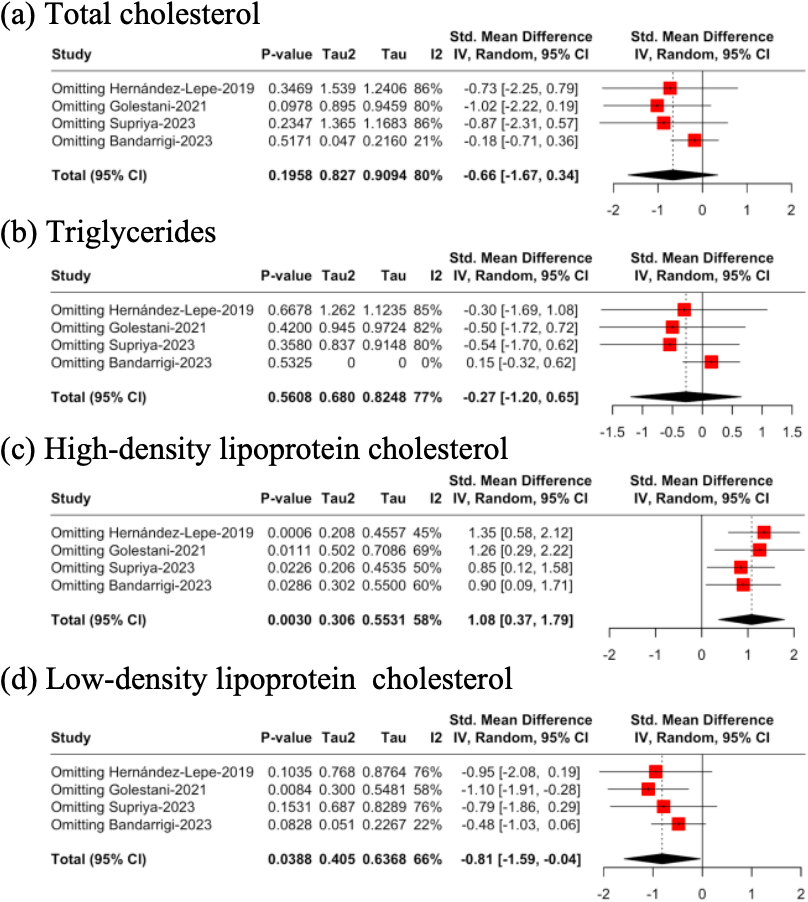


**Supplementary material 13**: Subgroup analyses of Spirulina versus control effects on total cholesterol

| **Subgroup** | **K(N)** | **Hedges' g** | **95%CI** | **p_d_** | **I^2^** | **Power** | **p_b_** |
| --- | --- | --- | --- | --- | --- | --- | --- |
| **Age(years)** |  |  |  |  |  |  |  |
| 18-44 | 5(299) | -0.87 | (-1.52, -0.21) | 0.01 | 83% | 82% | 0.04 |
| 45-59 | 6(208) | -0.98 | (-1.52, -0.44) | <0.01 | 68% | 95% |  |
| >60 | 2(53) | -0.04 | (-0.58, 0.50) | 0.88 | 0% | 4% |  |
| **Baseline BMI**  **(kg/m^2^)** |  |  |  |  |  |  |  |
| 25-30 | 8(370) | -0.87 | (-1.44, -0.29) | <0.01 | 82% | 91% | 0.46 |
| >30 | 5(190) | -0.62 | (-0.96, -0.27) | <0.01 | 23% | 93% |  |
| **Health Condition** |  |  |  |  |  |  |  |
| None | 6(193) | -0.40 | (-0.75, -0.05) | 0.03 | 29% | 62% | <0.01 |
| T2DM | 3(77) | -1.26 | (-1.77, -0.75) | <0.01 | 0% | 99% |  |
| HTN | 2(91) | -1.18 | (-1.95, -0.41) | <0.01 | 65% | 83% |  |
| HIV-ART-naïve | 1(159) | -1.61 | (-1.97, -1.26) | N/A | N/A | N/A |  |
| MS | 1(40) | 0.08 | (-0.54, 0.70) | N/A | N/A | N/A |  |
| **Spirulina Forms** |  |  |  |  |  |  |  |
| Tablet | 5(171) | -0.80 | (-1.28, -0.32) | <0.01 | 52% | 91% | <0.01 |
| Capsule | 4(116) | -0.66 | (-1.25, -0.08) | 0.03 | 54% | 65% |  |
| Power | 2(200) | -1.61 | (-1.93, -1.29) | <0.01 | 0% | 100% |  |
| liquid | 1(40) | 0.08 | (-0.54, 0.70) | N/A | N/A | N/A |  |
| Not specified | 1(33) | -0.19 | (-0.87, 0.50) | N/A | N/A | N/A |  |
| **Dose(g/day)** |  |  |  |  |  |  |  |
| <2 | 3(116) | -0.09 | (-0.45, 0.28) | 0.64 | 0% | 6% | <0.01 |
| 2 | 5(176) | -0.96 | (-1.39, -0.53) | <0.01 | 42% | 99% |  |
| 4-10 | 5(268) | -1.08 | (-1.70, -0.46) | <0.01 | 75% | 97% |  |
| **Duration(weeks)** |  |  |  |  |  |  |  |
| ≤8 | 6(162) | -0.97 | (-1.51, -0.44) | <0.01 | 59% | 94% | 0.43 |
| ≥12 | 7(398) | -0.66 | (-1.21, -0.11) | 0.02 | 84% | 73% |  |

K: number of studies; N: number of participants; Hedges’ g: effect size; 95% CI: 95% confidence interval; p_d_: significance of pooled effect; I^2^: heterogeneity; Power: statistical power; p_b_: significance between subgroups; None: no health conditions other than overweight/obesity; T2DM: type 2 diabetes mellitus; HTN: hypertension; HIV-ART-naïve: HIV-infected, antiretroviral therapy-naïve; MS: metabolic syndrome; N/A: Not Applicable

**Supplementary material 14**: Subgroup analyses of Spirulina versus control effects on triglycerides

| **Subgroup** | **K(N)** | **Hedges' g** | **95%CI** | **p_d_** | **I^2^** | **Power** | **p_b_** |
| --- | --- | --- | --- | --- | --- | --- | --- |
| **Age(years)** |  |  |  |  |  |  |  |
| 18-44 | 5(299) | -0.39 | (-1.14, 0.36) | 0.31 | 88% | 29% | 0.16 |
| 45-59 | 6(208) | -0.93 | (-1.36, -0.50) | <0.01 | 51% | 99% |  |
| >60 | 2(53) | -0.30 | (-0.85, 0.24) | 0.27 | 0% | 15% |  |
| **Baseline BMI**  **(kg/m^2^)** |  |  |  |  |  |  |  |
| 25-30 | 8(370) | -0.98 | (-1.35, -0.61) | <0.01 | 56% | 100% | <0.01 |
| >30 | 5(190) | -0.10 | (-0.39, 0.18) | 0.48 | 0% | 8% |  |
| **Health Condition** |  |  |  |  |  |  |  |
| None | 6(193) | -0.11 | (-0.39, 0.17) | 0.45 | 0% | 9% | <0.01 |
| T2DM | 3(77) | -1.22 | (-1.73, -0.72) | <0.01 | 0% | 99% |  |
| HTN | 2(91) | -0.83 | (-1.93, 0.26) | 0.14 | 84% | 50% |  |
| HIV-ART-naïve | 1(159) | -1.40 | (1.75, -1.06) | N/A | N/A | N/A |  |
| MS | 1(40) | -0.58 | (-1.22, 0.05) | N/A | N/A | N/A |  |
| **Spirulina Forms** |  |  |  |  |  |  |  |
| Tablet | 5(171) | -0.64 | (-1.32, 0.04) | 0.07 | 76% | 55% | <0.01 |
| Capsule | 4(116) | -0.32 | (-0.68, 0.05) | 0.09 | 0% | 33% |  |
| Power | 2(200) | -1.41 | (-1.72, -1.09) | <0.01 | 0% | 100% |  |
| liquid | 1(40) | -0.58 | (-1.22, 0.05)) | N/A | N/A | N/A |  |
| Not specified | 1(33) | -0.21 | (-0.90, 0.47) | N/A | N/A | N/A |  |
| **Dose(g/day)** |  |  |  |  |  |  |  |
| <2 | 3(116) | -0.24 | (-0.76, 0.29) | 0.38 | 46% | 21% | 0.32 |
| 2 | 5(176) | -0.71 | (-1.24, -0.18) | <0.01 | 63% | 78% |  |
| 4-10 | 5(268) | -0.78 | (-1.41, -0.15) | 0.02 | 78% | 81% |  |
| **Duration(weeks)** |  |  |  |  |  |  |  |
| ≤8 | 6(162) | -1.00 | (-1.40, -0.59) | <0.01 | 28% | 100% | 0.07 |
| ≥12 | 7(398) | -0.39 | (-0.91, 0.13) | 0.14 | 83% | 40% |  |

**Supplementary material 15**: Subgroup analyses of Spirulina versus control effects on high-density lipoprotein cholesterol

| **Subgroup** | **K(N)** | **Hedges' g** | **95%CI** | **p_d_** | **I^2^** | **Power** | **p_b_** |
| --- | --- | --- | --- | --- | --- | --- | --- |
| **Age(years)** |  |  |  |  |  |  |  |
| 18-44 | 5(299) | 0.63 | (-0.33, 1.60) | 0.2 | 92% | 40% | 0.29 |
| 45-59 | 6(208) | 0.67 | (0.20, 1.13) | 0.01 | 60% | 83% |  |
| >60 | 2(53) | -0.25 | (-1.29, 0.80) | 0.64 | 71% | 20% |  |
| **Baseline BMI**  **(kg/m^2^)** |  |  |  |  |  |  |  |
| 25-30 | 8(370) | 0.66 | (-0.05, 1.38) | 0.07 | 89% | 56% | 0.33 |
| >30 | 5(190) | 0.26 | (-0.13, 0.64) | 0.19 | 41% | 30% |  |
| **Health Condition** |  |  |  |  |  |  |  |
| None | 6(193) | 0.08 | (-0.37, 0.52) | 0.73 | 56% | 10% | <0.01 |
| T2DM | 3(77) | 0.67 | (0.20, 1.15) | <0.01 | 0% | 72% |  |
| HTN | 2(91) | 0.96 | (-0.10, 2.03) | 0.08 | 82% | 58% |  |
| HIV-ART-naïve | 1(159) | 1.87 | (1.50, 2.25) | N/A | N/A | N/A |  |
| MS | 1(40) | 0.00 | (-0.62, 0.62) | N/A | N/A | N/A |  |
| **Spirulina Forms** |  |  |  |  |  |  |  |
| Tablet | 5(171) | 0.31 | (-0.16, 0.77) | 0.20 | 52% | 33% | <0.01 |
| Capsule | 4(116) | 0.53 | (0.16, 0.90) | <0.01 | 0% | 72% |  |
| Power | 2(200) | 1.80 | (1.47, 2.13) | <0.01 | 0% | 100% |  |
| liquid | 1(40) | 0.00 | (-0.62, 0.62) | N/A | N/A | N/A |  |
| Not specified | 1(33) | -0.75 | (-1.46, -0.04) | N/A | N/A | N/A |  |
| **Dose(g/day)** |  |  |  |  |  |  |  |
| <2 | 3(116) | -0.04 | (-0.40, 0.33) | 0.83 | 0% | 4% | 0.06 |
| 2 | 5(176) | 0.72 | (0.15, 1.29) | 0.01 | 68% | 74% |  |
| 4-10 | 5(268) | 0.62 | (-0.40, 1.64) | 0.24 | 92% | 36% |  |
| **Duration(weeks)** |  |  |  |  |  |  |  |
| ≤8 | 6(162) | 0.78 | (0.37, 1.20) | <0.01 | 35% | 95% | 0.32 |
| ≥12 | 7(398) | 0.33 | (-0.44, 1.11) | 0.40 | 92% | 21% |  |

**Supplementary material 16**: Subgroup analyses of Spirulina versus control effects on low-density lipoprotein cholesterol

| **Subgroup** | **K(N)** | **Hedges' g** | **95%CI** | **p_d_** | **I^2^** | **Power** | **p_b_** |
| --- | --- | --- | --- | --- | --- | --- | --- |
| **Age(years)** |  |  |  |  |  |  |  |
| 18-44 | 5(299) | -0.66 | (-1.37, 0.06) | 0.07 | 86% | 58% | 0.28 |
| 45-59 | 6(208) | -0.94 | (-1.58, -0.29) | <0.01 | 78% | 84% |  |
| >60 | 2(53) | -0.12 | (-0.89, 0.64) | 0.75 | 46% | 11% |  |
| **Baseline BMI**  **(kg/m^2^)** |  |  |  |  |  |  |  |
| 25-30 | 8(370) | -0.84 | (-1.45, -0.23) | <0.01 | 84% | 86% | 0.33 |
| >30 | 5(190) | -0.48 | (-0.89, -0.06) | 0.02 | 28% | 75% |  |
| **Health Condition** |  |  |  |  |  |  |  |
| None | 6(193) | -0.26 | (-0.61, 0.08) | 0.14 | 28% | 33% | <0.01 |
| T2DM | 3(77) | -1.04 | (-1.54, -0.55) | <0.01 | 0% | 96% |  |
| HTN | 2(91) | -1.40 | (-2.48, -0.32) | 0.01 | 80% | 77% |  |
| HIV-ART-naïve | 1(159) | -1.51 | (-1.86, -1.15) | N/A | N/A | N/A |  |
| MS | 1(40) | 0.31 | (-0.32, 0.93) | N/A | N/A | N/A |  |
| **Spirulina Forms** |  |  |  |  |  |  |  |
| Tablet | 5(171) | -0.62 | (-1.15, -0.08) | 0.02 | 63% | 0.68 | <0.01 |
| Capsule | 4(116) | -0.76 | (-1.14, -0.39) | <0.01 | 0% | 94% |  |
| Power | 2(200) | -1.62 | (-2.01, -1.23) | <0.01 | 18% | 100% |  |
| liquid | 1(40) | 0.31 | (-0.32, 0.93) | N/A | N/A | N/A |  |
| Not specified | 1(33) | 0.21 | (-0.47, 0.90) | N/A | N/A | N/A |  |
| **Dose(g/day)** |  |  |  |  |  |  |  |
| <2 | 3(116) | 0.06 | (-0.37, 0.48) | 0.79 | 22% | 7% | <0.01 |
| 2 | 5(176) | -1.08 | (-1.62, -0.55) | <0.01 | 61% | 97% |  |
| 4-10 | 5(268) | -0.76 | (-1.43, -0.09) | 0.03 | 81% | 74% |  |
| **Duration(weeks)** |  |  |  |  |  |  |  |
| ≤8 | 6(162) | -1.11 | (-1.53, -0.68) | <0.01 | 34% | 100% | 0.07 |
| ≥12 | 7(398) | -0.42 | (-1.03, 0.19) | 0.18 | 87% | 37% |  |

**Supplementary material 17**: Linear regression plots of participant characteristics and Spirulina supplementation protocols versus blood lipid outcomes in Spirulina versus control


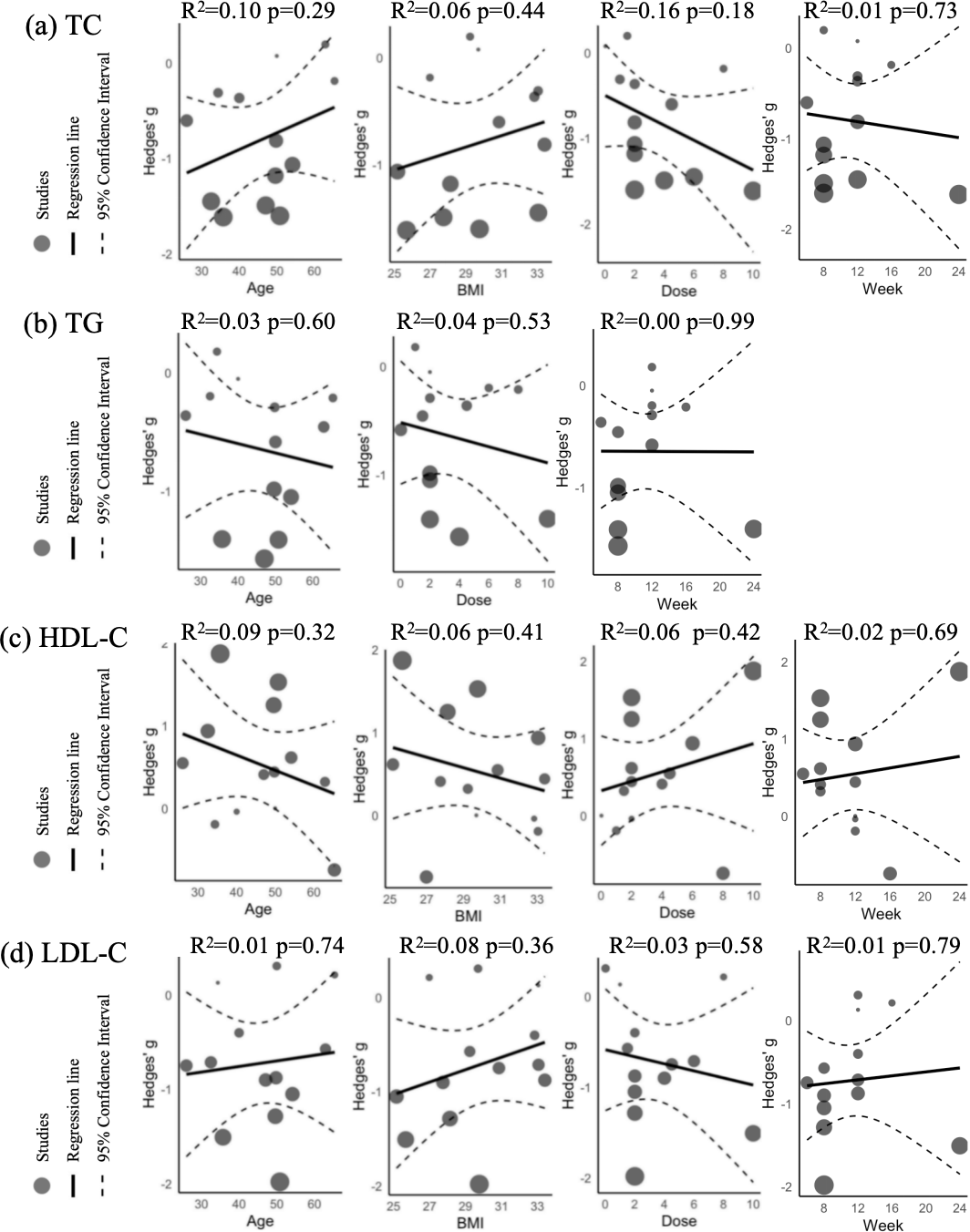


TC: total cholesterol; TG: triglycerides; HDL-C: high-density lipoprotein cholesterol; LDL-C: low-density lipoprotein cholesterol; R^2^: model fit; p: significance of the regression line; Age: participant age; BMI: baseline body mass index; Dose: Spirulina dose (g/day); Duration: intervention duration (weeks)

**Supplementary material 18**: Non-linear regression plots of Spirulina supplementation protocols versus blood lipid outcomes in Spirulina versus control


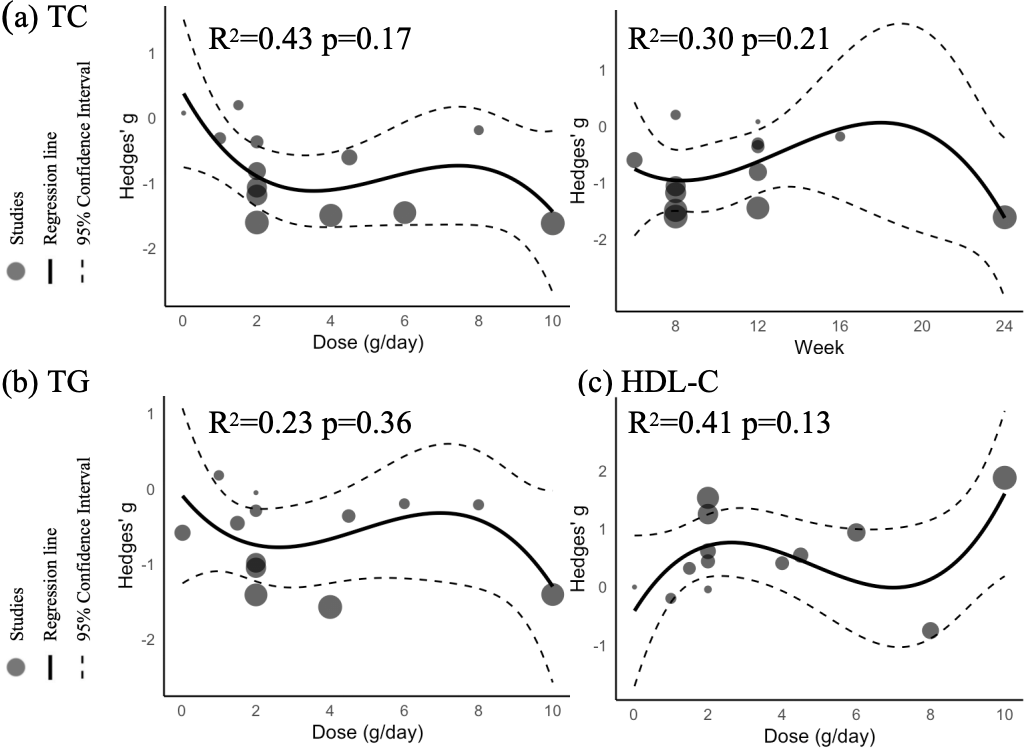


**Supplementary material 19**: Risk of bias assessment for included studies


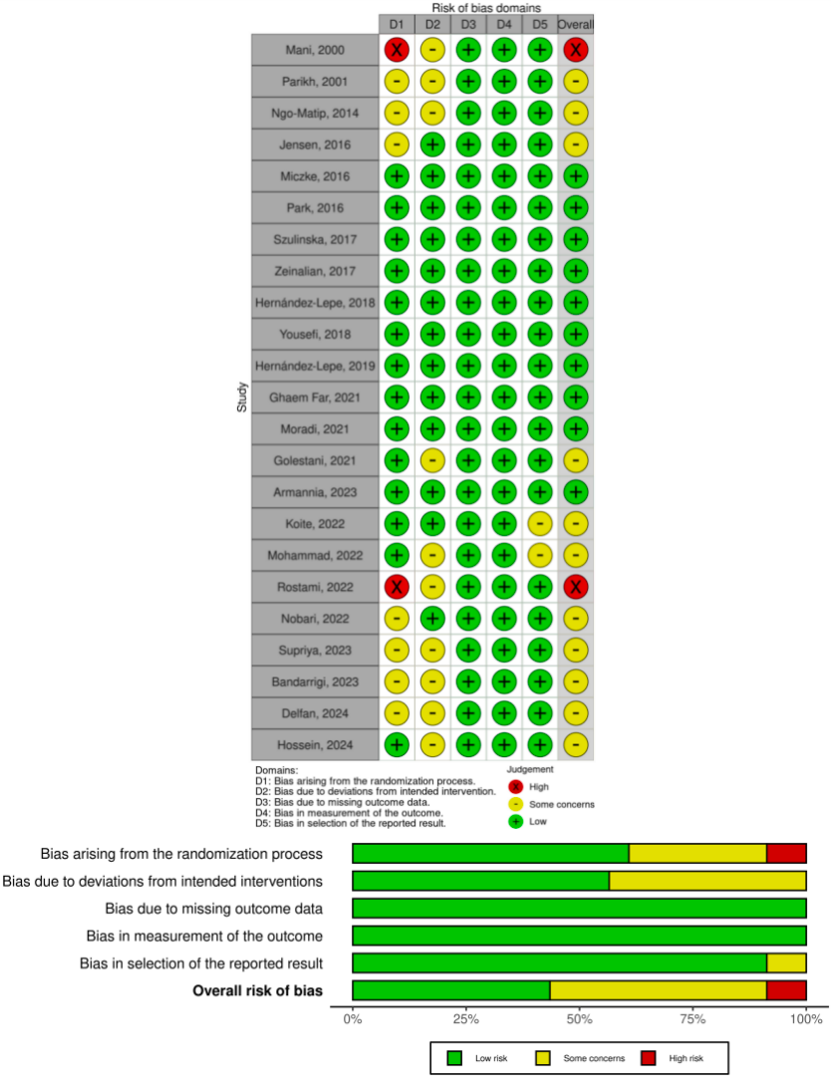


**Supplementary material 20**: Funnel plot of studies included in Spirulina versus control analyses


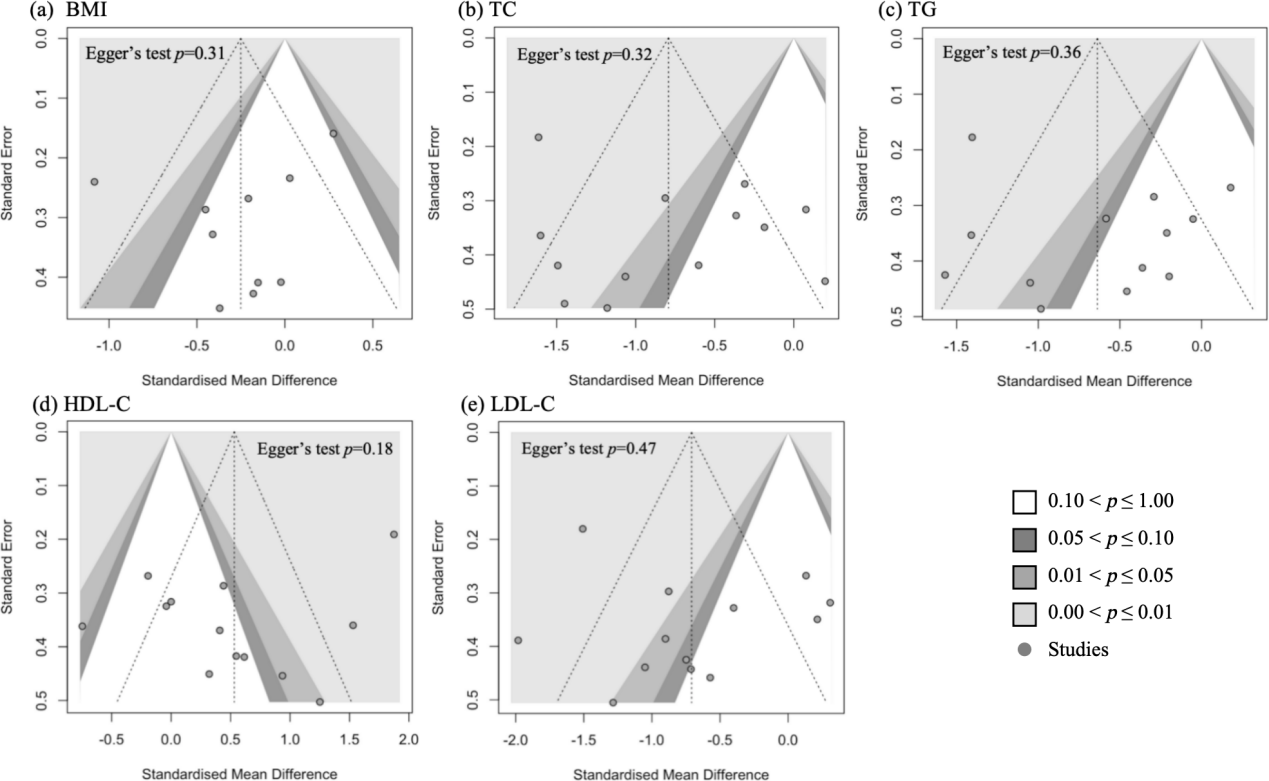

Supplement: Supplementary file 1 [file Data_Sheet_1.docx]
